# Protein Kinase STK24 Promotes Tumor Immune Evasion via the AKT‐PD‐L1 Axis

**DOI:** 10.1002/advs.202304342

**Published:** 2024-01-16

**Authors:** Ning Wang, Yu Jiang, Mengjie Li, Haofei Wang, Jie Pan, Yang Tang, Shaofang Xie, Yunyang Xu, Xu Li, Xuefei Zhou, Pinglong Xu, Wenlong Lin, Xiaojian Wang

**Affiliations:** ^1^ Institute of Immunology and Bone Marrow Transplantation Center The First Affiliated Hospital School of Medicine Zhejiang University Hangzhou Zhejiang 310058 China; ^2^ Department of Clinical Laboratory Second Affiliated Hospital of Zhejiang University School of Medicine Hangzhou Zhejiang 310058 China; ^3^ Westlake Laboratory of Life Sciences and Biomedicine School of Life Sciences Westlake University Hangzhou Zhejiang 310024 China; ^4^ Department of Pharmacology School of Medicine Zhejiang University Hangzhou Zhejiang 310058 China; ^5^ Life Sciences Institute Zhejiang University Hangzhou Zhejiang 310058 China

**Keywords:** AKT, programmed cell death ligand 1 (PD‐L1), serine/threonine protein kinase 24 (STK24), tumor immune evasion

## Abstract

Immunotherapy targeting PD‐L1 is still ineffective for a wide variety of tumors with high unpredictability. Deploying combined immunotherapy with alternative targeting is practical to overcome this therapeutic resistance. Here, the deficiency of serine‐threonine kinase STK24 is observed in tumor cells causing substantial attenuation of tumor growth in murine syngeneic models, a process relying on cytotoxic CD8^+^ T and NK cells. Mechanistically, STK24 in tumor cells associates with and directly phosphorylates AKT at Thr21, which promotes AKT activation and subsequent PD‐L1 induction. Deletion or inhibition of STK24, by contrast, blocks IFN‐γ‐mediated PD‐L1 expression. Various murine models indicate that in vivo silencing of STK24 can significantly enhance the efficacy of the anti‐PD‐1 blockade strategy. Elevated STK24 levels are observed in patient specimens in multiple tumor types and inversely correlated with intratumoral infiltration of cytotoxic CD8^+^ T cells and with patient survival. The study collectively identifies STK24 as a critical modulator of antitumor immunity, which engages in AKT and PD‐L1/PD‐1 signaling and is a promising target for combined immunotherapy.

## Graphical abstract

Immunotherapy targeting PD‐L1 is ineffective in many tumors and with inherent unpredictability. Substantial efforts are being channeled toward identifying novel targets to enhance immunotherapy efficacy through combination treatments. Here, STK24 is identified as a promoter of tumorigenesis in a non‐cancer cell‐autonomous manner. STK24 phosphorylates AKT at a previously unrecognized Thr21, augments PD‐L1 expression, and facilitates the evasion against antitumor immunity.

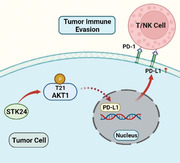

## 1 Introduction

Interactions between tumor cells and the host immune microenvironment, particularly involving infiltrating immune cells, constitute a critical determinant of disease progression, metastasis, and recurrence. Immune checkpoint blockades, targeting cytotoxic T‐lymphocyte‐associated protein 4 (CTLA‐4), programmed cell death protein 1 (PD‐1), or programmed cell death ligand 1 (PD‐L1), have exhibited notable efficacy in a defined subset of cancer patients, encompassing non‐small cell lung cancer (NSCLC), metastatic melanoma, and microsatellite instability tumors.^[^ ^1^ ^]^ However, the broad clinical success of immune activation treatment remains limited. The majority of patients exhibit poor response due to tumor immune escape and resistance to α‐PD‐1/PD‐L1 therapy.^[^ ^2^ ^]^

Tumor progression and immunotherapy‐refractory tumors have been extensively investigated from various aspects such as tumor cell proliferation, apoptosis, and neoantigen production.^[^ ^3^ ^]^ Recent studies, however, brought to light that genetic and epigenetic alterations in tumors give rise to weakened immune surveillance within the tumor microenvironment (TME), facilitating the onset and progression of various cancer types.^[^ ^4^ ^]^ Oncogene MYC plays critical roles in cell proliferation and growth, emerging evidence highlights that MYC regulates PD‐L1 and CD47 expression in tumor cells and remodels the TME, thereby allowing tumor immune escape.^[^ ^5^ ^]^ Moreover, a discernible enhancement in immunotherapeutic efficacy against tumors has been demonstrated through the strategic combination of drugs targeting EGFR, KRAS, or MYC with anti‐PD‐1/PD‐L1 agents.^[^ ^5^, ^6^ ^]^ These results prompted many current efforts to identify novel factors that modulate tumor immune response, which is critically important in advancing precision cancer immunotherapy.

The serine‐threonine kinase STK24 belongs to the germinal center kinase (GCK) III subfamily of the Sterile‐20 (Ste20) family that primarily involves the activation of mitogen‐activated protein kinase (MAPK) cascades that govern diverse cellular functions, including apoptosis and cell migration.^[^ ^7^ ^]^ We recently reported that STK24 positively regulates IL‐17R‐mediated inflammation responses, by promoting the binding between TAK1 and IKKβ in the context of autoimmune disease.^[^ ^8^ ^]^ In addition, we revealed that STK24 regulates high‐fat diet (HFD)‐induced metabolic disorders by disrupting the NLRP3 inflammasome.^[^ ^9^ ^]^ It is reported that STK24 promotes tumorigenicity via regulating the VAV2/Rac1 signaling axis in breast cancer cells,^[^ ^10^ ^]^ or regulating the expression of P21 in gastric cancer cells.^[^ ^11^ ^]^ Cao et al. recently found that STK24 promotes the proliferation of non‐small cell lung cancer cells via stabilizing STAT3.^[^ ^12^ ^]^ However, STK24 knockdown promotes tumorigenesis in gastric cancer animal models.^[^ ^13^ ^]^ Thus, STK24 appears to have pleiotropic roles in tumorigenesis. In tumor cell surveillance, IFN‐γ secreted by immune cells promotes tumor cell‐clearing role in the tumor microenvironment; IFN‐γ also upregulates the expression of PD‐L1 in tumor cells and affects the efficacy of immunotherapy via STAT1/STAT3 signaling pathway.^[^ ^14^ ^]^ Although STK24 has been reported to regulate the STAT3 pathway,^[^ ^12^ ^]^ its function in tumor immunity remains unclear.

In this study, we found that STK24 promotes tumorigenesis by compromising tumor immunity. STK24‐defect has no detectable effect on tumor formation in immunodeficient mice, but significantly impedes tumor growth in immunocompetent mice, accompanied by increased infiltration of cytotoxic CD8^+^ T cells and natural killer (NK) cells in tumor tissues. We further identified that STK24 augments PD‐L1 expression in tumor cells via phosphorylating AKT at a previously unrecognized Thr21 residue. As a result, the STK24 deficiency significantly boosts the efficacy of anti‐PD‐1 immunotherapy. Moreover, STK24 expression levels positively correlate with AKT Thr21 phosphorylation and PD‐L1 expression, inversely correlate with the infiltration of CD8^+^ T and patient survival outcomes.

## 2 Results

### 2.1 STK24 Expression is Upregulated in Tumor Specimens and Correlated with Poor Prognosis

**Figure 1:**
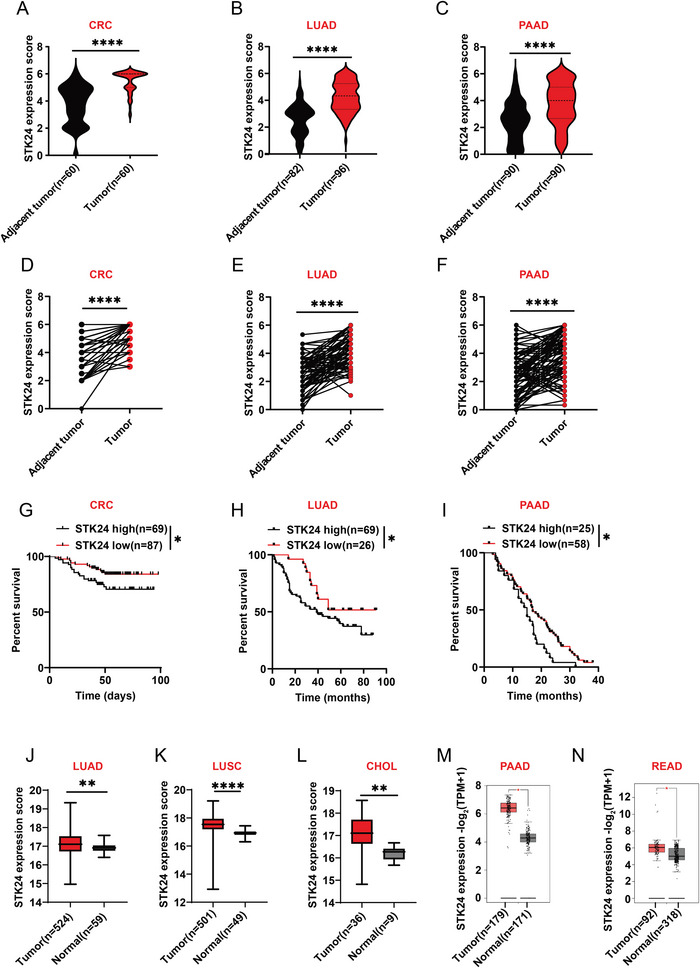
STK24 expression is upregulated in multiple cancer types and correlated with poor prognosis. A–C) Statistical analysis of STK24 protein expression levels determined by IHC staining of the adjacent‐tumor tissues and tumor tissues from the patients with colorectal cancer (CRC) (A), lung adenocarcinoma (LUAD) (B), or pancreatic adenocarcinoma (PAAD) (C). D–F) Statistical analysis of STK24 protein expression levels determined by IHC staining of the adjacent‐tumor tissues and tumor tissues from the same patient with CRC ((D) *n* = 60), LUAD ((E) *n* = 81), or PAAD ((F) *n* = 90). G–I) Kaplan–Meier curves of overall survival in the set of patients with G) CRC, H) LUAD, I) or PAAD based on STK24 protein expression level detected in the tumor tissues. J–L) Comparison of STK24 gene expression levels in tumor tissues or normal tissues from patients with LUAD (J), lung squamous cell carcinoma (LUSC) (K), and cholangio carcinoma (CHOL) (L) based on the HPA database. M,N) Comparison of STK24 expression levels in tumor tissues or normal tissues from patients with pancreatic adenocarcinoma (PAAD) (M) and rectum adenocarcinoma (READ) (N) based on the GEPIA database. Results are presented as mean ± SEM. ^*^ *p* <0.05, ^**^ *p* <0.01, ^****^ *p* <0.0001. P values were calculated by unpaired Student's *t*‐tests in (A–F) and (J–L) and log‐rank test in (G–I). See also Figure S1 (Supporting Information).

To determine the role of STK24 in tumorigenesis, we analyzed STK24 expression in three groups of patient specimen, including colorectal cancer (CRC), lung adenocarcinoma (LUAD), and pancreatic adenocarcinoma (PAAD) by tissue microarray (TMA)‐based immunohistochemistry (IHC). Quantification of the IHC results indicated a substantial increase in STK24 protein expression within tumor tissues compared to corresponding tumor margins in CRC (**Figure** **1**A; Figure S1A, Supporting Information), LUAD (Figure 1B; Figure S1B, Supporting Information), and PAAD patients (Figure 1C; Figure S1C, Supporting Information). Similarly, elevated STK24 protein expression in tumor tissues was observed when compared to matched adjacent tumor tissue controls in most of individual cases (Figure 1D–F). Tumor tissues were then divided into groups with high or low levels of STK24 according to IHC scores, patients with high STK24 protein expression exhibited shorter overall survival (Figure 1G–I). Collectively, these findings strongly indicate that STK24 expression levels exhibit a consistent elevation across diverse cancer types, thereby correlating with poor outcomes.

To validate the observations from the patient cohorts, we analyzed *STK24* expression status using Tumor Immune Estimation Resource (TIMER) databases, The Human Protein Atlas (HPA), and Gene Expression Profiling Interactive Analysis (GEPIA) databases. Examination of TIMER unveiled a significant upregulation of *STK24* gene expression in various kinds of tumors, compared with corresponding normal tissues (Figure S1D, Supporting Information). Consistently, we observed the enhanced *STK24* gene expression in LUAD, lung squamous cell carcinoma (LUSC), and cholangiocarcinoma (CHOL) (Figure 1J–L) in HPA data set. A similarly enhanced *STK24* gene expression pattern is also seen in PAAD and rectum adenocarcinoma (READ) from the GEPIA data set (Figure 1M,N). Furthermore, patients with higher *STK24* gene expression show decreased overall survival than those with lower *STK24* gene expression in nine cohorts from the HPA database (Figure S1E–M, Supporting Information). Together, these results strongly suggest that STK24 is broadly upregulated across many cancer types and is indicative of poor prognosis.

### 2.2 Ablation of STK24 Attenuates Tumor Growth in Immunocompetent Mice

To examine the functional role of *Stk24* in tumor cells, we generated *Stk24* knockout mutants by CRISPR‐Cas9 targeting or *Stk24* knockdown cell lines by siRNA or small hairpin RNAs (shRNAs) in diverse mouse cell lines. These include colon cancer cells CT26, MC38, lung cancer cells LLC, and pancreatic cancer cells KPC. Of note is that the loss of *Stk24* (*Stk24* KO) showed no detectable cell proliferation defect compared to isogenic wild‐type control cells (*Ctrl*) (Figure S2A–D, Supporting Information). Consistently, knockdown of *Stk24* in CT26, MC38, LLC, and KPC cells did not alter in vitro growth rates in these tumor cells (Figure S2E–H, Supporting Information). Similarly, knockout or knockdown of *Stk24* gene failed to alter cultured cell proliferation in human colon cancer cell line HCT116, lung cancer cell line NCI‐H1299, and A549 (Figure S2I–L, Supporting Information). These results suggest that STK24 function is not required for cell growth per se.

**Figure 2:**
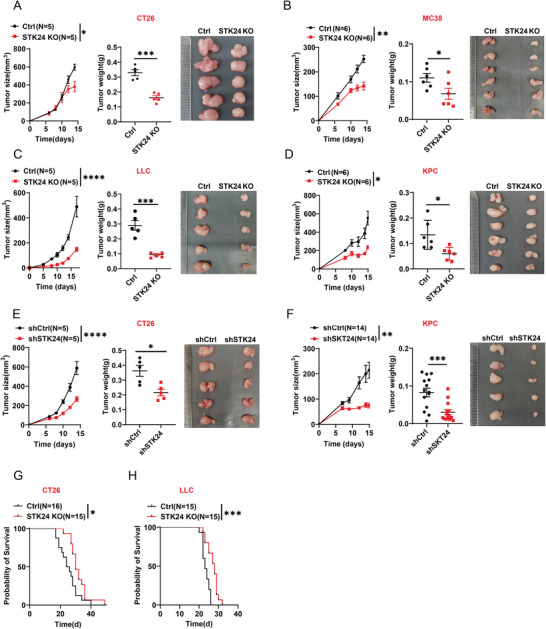
STK24 deficiency in tumor cells attenuates tumor growth in syngeneic mice. A) *Ctrl* and *Stk24* KO CT26 cells (5 × 10^5^) were subcutaneously transplanted into BALB/c mice. Tumor growth curves (left), tumor weight (middle), and representative tumor images (right) were shown. B–D) *Ctrl* and *Stk24* KO MC38 cells (5 × 10^5^) (B), C) LLC cells (1 × 10^6^), D) or KPC cells (1 × 10^6^) were subcutaneously transplanted into C57BL/6 mice. E,F) Sh*Ctrl* and sh*Stk24*‐transfected tumor cell were subcutaneously transplanted into syngeneic mice. E) CT26 cells (5 × 10^5^). F) KPC cells (1 × 10^6^). G,H) Kaplan–Meier survival curves of mice subcutaneously transplanted with *Ctrl* and *Stk24* KO mouse tumor cells. G) CT26 cell lines, grown in BALB/c mice. H) LLC cell lines, grown in C57BL/6 mice. Results represent at least two independent experiments and are presented as mean ± SEM. Each dot represents a biological sample. ^*^ *p* <0.05, ^**^ *p* <0.01, ^***^ *p* <0.001, ^****^ *p* <0.0001. P values of tumor growth curves and tumor weight were calculated by two‐way ANOVA and unpaired Student's *t*‐tests, respectively, in (A–F). P values of Kaplan–Meier survival curves were calculated by log‐rank test in (G,H). See also Figure S2 (Supporting Information).

When CT26 or LLC *Stk24* KO mutant cells were inoculated into immunodeficient NOD‐SCID IL2rg^−/−^ (NSG) mice, the in vivo growth of the xenografted tumor cells was indistinguishable between the *Stk24* KO mutant and wild‐type controls (Figure S2M,N, Supporting Information). These results indicate that STK24 deficiency does not affect tumor cell proliferation in the absence of an immune environment. Subsequently, we tested tumor formation of the *Stk24* mutants in syngeneic immuno‐competent mice by inoculating mouse *Ctrl* or *Stk24* KO CT26 cells into BALB/c mice. As depicted (**Figure** **2**A), the *Stk24* knockout group exhibited a drastic decrease in tumor growth. Likewise, deletion of *Stk24* in MC38, LLC, and KPC cells of C57BL/6 origin led to significantly delayed tumor development in the immune‐competent C57BL/6 strain (Figure 2B–D), suggesting that STK24 function likely involves in tumor immune response, rather than cell proliferation per se.

Extending the above notion further, we performed subcutaneous tumor implantations using shRNA‐mediated *Stk24* knockdown cells and observed a substantial reduction of tumor growth in both *Stk24*‐depleted CT26 (Figure 2E) and KPC cells (Figure 2F). Moreover, prolonged survival was evident in the *Stk24*‐deficient CT26 (Figure 2G) and LLC groups (Figure 2H) of mice, compared with that of the control group.

Given that the role of STK24 in tumor growth likely pertains to immune response, our investigation aimed to clarify whether such a role affects the onset and development of primary tumors. Intraperitoneal *(i.p.)* injection of urethane induces lung tumorigenesis in mice accomplished with a single‐driver mutation in the *KRAS* gene and pathological features congruent with human adenocarcinomas.^[^ ^15^ ^]^ Thus, we induced primary lung tumors in *Stk24*‐deficient mice (*Stk24* ^h/h^) and wild‐type mice (WT) by intraperitoneal urethane injection. As shown in Figure S2O,P (Supporting Information), *Stk24* knockout significantly inhibited the formation of primary tumor nodules in the lung. Collectively, these results imply that STK24 is not involved in the growth regulation of the tumors but contributes to anti‐tumor immune response.

### 2.3 STK24 Deficiency Enhances the Intratumoral Infiltration of Cytotoxic CD8+T Cells and NK Cells

**Figure 3:**
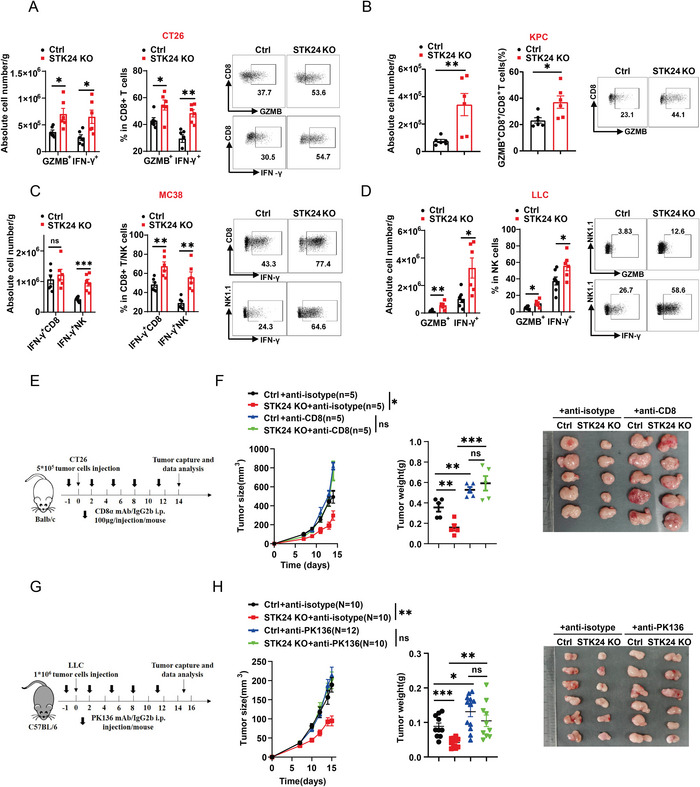
STK24 deficiency enhances intratumoral infiltration of cytotoxic T cells and NK cells. A,B) Quantify numbers (left), proportion (middle), and representative plots (right) of tumor‐infiltrating GZMB^+^CD8^+^T cells and IFN‐γ^+^ CD8^+^T cells in tumor tissues from *Ctrl* and *Stk24* KO CT26 tumor cells A) or KPC tumor cells B) subcutaneously inoculated mice models were determined by flow cytometry. C,D) Quantify numbers (left), proportion (middle), and representative plots (right) of tumor‐infiltrating activated CD8^+^T cells and NK cells in tumor tissues from *Ctrl* and *Stk24* KO MC38 tumor cells (C) or LLC tumor cells (D) subcutaneously inoculated mice models were determined by flow cytometry. E,F) BALB/c mice were implanted with 5 × 10^5^ *Ctrl* or *Stk24* KO CT26 cells, and then intraperitoneally injected with the anti‐CD8α mAb or the anti‐IgG isotype control (IgG2β) (100 µg) every three days. (E) Schematic diagram of the treatment plan. (F) Tumor growth curves (left), tumor weight (middle), and representative tumor images (right) of *Ctrl* and *Stk24* KO CT26 tumors were shown. G,H) C57BL/6 mice were implanted with 1×10^6^ *Ctrl* or *Stk24* KO LLC cells and received PK136 Ab treatment or IgG isotype control (IgG2β). Results represent at least two independent experiments and are presented as mean ± SEM. Each dot represents a biological sample. ns, no significant difference. ^*^ *p* <0.05, ^**^ *p* <0.01, ^***^ *p* <0.001, ^****^ *p* <0.0001. P values of tumor growth curves were calculated by two‐way ANOVA in (F,H). P values in (A–D) and P values of tumor weight in (F,H) were calculated by unpaired Student's *t*‐tests. See also Figure S3 (Supporting Information).

To determine the mechanisms underpinning the anti‐tumor immune response mediated by STK24, we examined immune cell profiles in both control and *Stk24*‐deficient CT26 or KPC tumors. Flow cytometry analyses revealed that the activity (IFN‐γ^+^ or GZMB^+^) of infiltrated CD8^+^ T cells was significantly increased in the *Stk24* KO group (**Figure** **3**A,B), while the composition of other immune cells remained unaffected (Figure S3A,B, Supporting Information). In MC38 cell‐inoculated mice, *Stk24* deficiency boosted the proportion of active IFN‐γ^+^ CD8^+^ T cells and IFN‐γ^+^ NK cells in the tumor tissues (Figure 3C; Figure S3C, Supporting Information). Likewise, *Stk24*‐deficient LLC tumors showed elevated infiltrations of GZMB^+^ and IFN‐γ^+^ NK cells in comparison to the *Stk24* ^+/+^ wild‐type control (Figure 3D; Figure S3D, Supporting Information). To verify whether STK24 in tumor cells could directly affect CD8^+^ T antigen‐specific killing ability, we co‐cultured OT1‐CD8^+^ cells with *Stk24* knockdown MC38‐OVA or KPC‐OVA cells. As shown Figure S3E,F (Supporting Information), *Stk24* deficiency in tumor cell directly rendered the tumor cells more susceptible to be killed by cytotoxic T lymphocytes (CTLs).

To dissect the anti‐tumor effect of STK24 deficiency in CT26 cells, we inoculated *Ctrl* and *Stk24* KO CT26 cells into immune‐competent BALB/c mice and administered continuous intraperitoneal injections of anti‐CD8α monoclonal neutralizing antibody (mAb) or anti‐immunoglobulin G (IgG) isotype control (IgG2β) during the tumorigenesis (Figure 3E). In mice inoculated with *Ctrl* CT26 cells, anti‐CD8α mAb treatment significantly promoted tumor burden compared with that of anti‐IgG2β treatment. Importantly, the difference in tumor growth between the *Ctrl* and *Stk24* KO groups disappeared after anti‐CD8α mAb treatment (Figure 3F), indicating that STK24 supports CT26 tumor development through inhibition of CD8^+^T cell‐dependent cytotoxic T cell responses. On the other hand, STK24 deficiency facilitated the activation of NK cells in LLC tumors (Figure 3D). Clearing NK cells with the anti‐PK136 antibody yielded no statistically significant tumor growth difference between the *Ctrl* and *Stk24* KO LLC tumors (Figure 3G,H), suggesting that the anti‐tumor effect of STK24 deletion primarily depends on NK cells in the LLC tumor‐bearing model.

### 2.4 STK24 Inhibits Anti‐Tumor Immunity by Promoting PD‐L1 Expression

**Figure 4:**
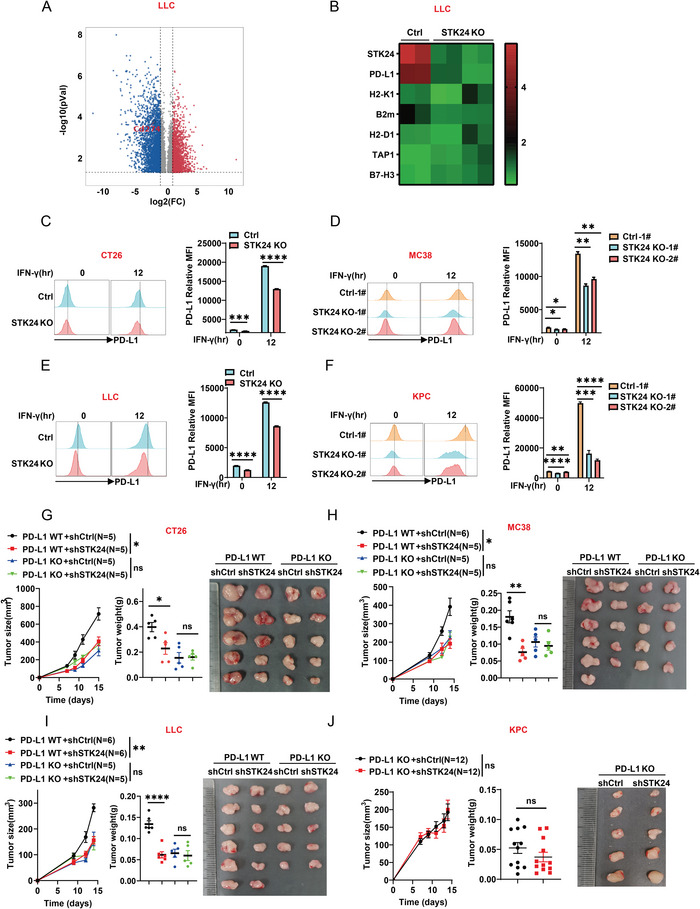
STK24 deficiency promotes tumor immune response by impairing PD‐L1 expression. A) Volcano plot from RNA‐seq analysis of *Ctrl* and *Stk24* KO LLC tumor cells. B) Heat map from RNA‐seq analysis of immune checkpoints related gene expression in *Ctrl* and *Stk24* KO LLC tumor cells. C–F) *Stk24* was knockout in mouse C) CT26, D) MC38, E) LLC, and F) KPC tumor cells by CRISPR‐Cas9, and the protein expression levels of PD‐L1 on the tumor cell surface was detected by flow cytometry with or without IFN‐γ (20 ng mL^−1^) treatment. FACS (left) and MFI (right) of PD‐L1^+^ membrane expression were shown. *n* = 3 biologically independent samples per group. G–J) sh*Ctrl* and sh*Stk24*‐transfected *Pd‐l1* KO CT26 (G), H) MC38, I) LLC, and J) KPC cells were subcutaneously transplanted into mice. Each dot represents a biological sample. Results represent at least two independent experiments and are presented as mean ± SEM. ns, no significant difference. ^*^ *p* <0.05, ^**^ *p* <0.01, ^***^ *p* <0.001, ^****^ *p* <0.0001. P values of tumor growth curves were calculated by two‐way ANOVA in (G–J). P values in (C–F) and P values of tumor weight in (G–J) were calculated by unpaired Student's *t*‐tests. See also Figure S4 (Supporting Information).

To further explore how STK24 regulates tumor cell function to affect the tumor immune microenvironment, comparative RNA‐Seq analysis was performed between *Ctrl* and *Stk24* KO LLC tumor cells in‐house. As shown in **Figure** **4**A,B, the *PD‐L1* gene expression in the *Stk24* KO LLC cells was significantly lower than that in the *Ctrl* LLC cells. Similar expression profile of *PD‐L1* was obtained from the RNA‐seq analysis of CT26 cells (Figure S4A,B, Supporting Information). Furthermore, the downregulation of *PD‐L1* gene expression in CT26, MC38, LLC, and KPC cells defective in STK24 was validated by quantitative real‐time PCR analysis (Figure S4C, Supporting Information).

As an important immunosuppressive receptor ligand, PD‐L1 inhibits lymphocyte activation and promotes tumor immune escape by binding to its receptor PD‐1 on immune cells, including T cells and NK cells.^[^ ^16^ ^]^ IFN‐γ is widely believed to be the predominant stimulator contributing to the inducible PD‐L1 expression in TME.^[^ ^17^ ^]^ Therefore, PD‐L1 protein expression was examined in several STK24 knockout cancer cell lines with or without IFN‐γ treatment. Compared to the *Ctrl* cells, cell surface PD‐L1 on *Stk24* KO CT26 (Figure 4C), MC38 (Figure 4D), LLC (Figure 4E), and KPC cells (Figure 4F) exhibited noticeable reduction with or without IFN‐γ stimulation. Similarly, STK24 depletion by siRNA or shRNA inhibited PD‐L1 protein expression on the above four tumor cells stimulated with IFN‐γ (Figure S4D–K, Supporting Information). In human NCI‐H1299, A549, and HCT116 tumor cells, STK24 knockdown (Figure S4L, Supporting Information) or knockout (Figure S4M, Supporting Information) also inhibited IFN‐γ‐induced PD‐L1 expression. These results suggest that STK24 function is important in PD‐L1 expression.

To determine whether STK24‐dependent tumor immune escape is indeed mediated by PD‐L1, we constructed PD‐L1 knockout mutants (*Pd‐l1 KO*) in CT26, MC38, LLC, and KPC cells via CRISPR‐Cas9‐mediated gene disruption and subsequently knocked down endogenous STK24 expression with shRNA(sh*Stk24*)‐mediated gene silencing in these cells. The PD‐L1 knockout efficacy was validated by FACS analysis and sanger sequencing (Figure S4N–R, Supporting Information). The PD‐L1 knockout and STK24‐silenced tumor cells, the PD‐L1 knockout control‐silenced cells, and their corresponding *Pd‐l1* WT control cells (sh*Ctrl* and sh*Stk24*) were subcutaneously injected into the mice, respectively. As shown in Figure 4G–J, when PD‐L1 expression was knocked out in these tumor cells, tumor growth between the control (sh*Ctrl*) and STK24 silenced (sh*Stk24*) groups appeared indistinguishable. Collectively, these findings strongly indicate that STK24 mediates tumor immune escape in a tumor PD‐L1 expression‐dependent manner.

### 2.5 STK24 Regulates Tumor Immune Evasion by Directly Phosphorylating AKT at Thr21 Residue

**Figure 5:**
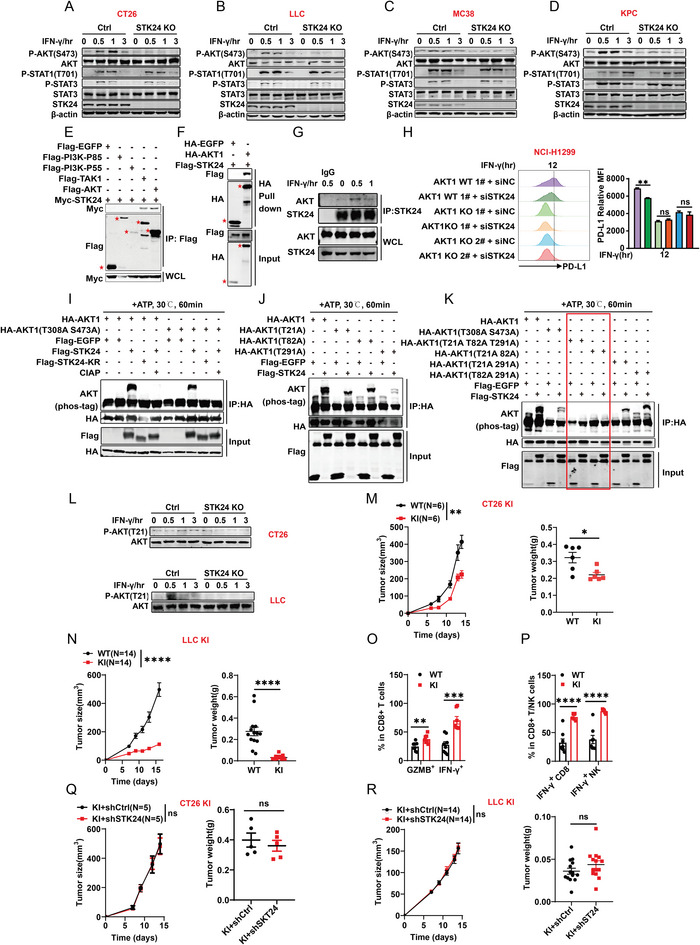
STK24 promotes PD‐L1 expression via phosphorylating AKT at T21 residue. A–D) Immunoblot analysis of AKT and JAK/STAT signal pathway with the indicated antibodies in *Stk24* knockout and wild‐type A) CT26, B) LLC, C) MC38, and D) KPC cell lines treated with IFN‐γ (100 ng mL^−1^) for indicated time. E) Immunoblot analysis of the interaction between AKT and STK24 with anti‐Flag immunoprecipitated in HEK293T cells. WCL: whole cell lysates. F) A HA‐tagged pull‐down experiment was performed with 1 µg fusion protein HA‐EGFP or HA‐AKT1 mixed with 2 µL pre‐cleared HA beads in 500 µL reaction medium, followed by the addition of 1 µg Flag‐STK24 recombinant protein and incubation at 4 °C for 4 h with gentle rotation. Pull‐down and input samples were analyzed by immunoblot with anti‐HA or anti‐Flag antibody. G) The endogenous complex of STK24 and AKT was detected by immunoprecipitation using anti‐STK24 antibody and analyzed by immunoblot by anti‐AKT or anti‐STK24 antibody in MC38 cells treated with IFN‐γ (100 ng mL^−1^) for indicated time. H) The expression of STK24 was knockdown in human *AKT1* KO NCI‐H1299 cells by siRNA, and the protein expression levels of PD‐L1 were detected by flow cytometry with IFN‐γ (20 ng mL^−1^) treatment for 12 h. *n* = 3 biologically independent samples per group. I) In vitro kinase assays of STK24 or STK24 mutant without kinase activity (KR), with AKT1 or AKT1 S473A T308A mutant as a substrate. The reaction samples were assessed by phos‐tag immunoblot analysis performed with anti‐AKT, anti‐HA, or anti‐Flag antibody. J) In vitro kinase assays of STK24 with AKT1, or AKT1 mutants, including AKT1‐T21A, AKT1‐T82A, or AKT1‐T291A as a substrate. K) In vitro kinase assays of STK24 with AKT1, or AKT1 mutants, including AKT1‐S473A T308A, AKT1‐T21A T82A T219A, AKT1‐T21A T82A, AKT1‐T21A T291A, or AKT1‐T82A T291A as a substrate. L) Immunoblot analysis of the phosphorylation of AKT T21 in STK24 knockout CT26 (upper) and LLC (down) cell lines treated with IFN‐γ (100 ng/ml) for indicated time. M,N) Wild‐type (WT) and AKT1 T21A knock‐in (KI) CT26 (M) or LLC (N) cells were subcutaneously transplanted into indicated mice. Each dot represents a biological sample. O,P) Quantify the proportion of tumor‐infiltrating activated CD8^+^T cells or NK cells of tumor tissue in WT or KI CT26 (O) or LLC (P) tumors were determined by flow cytometry. Each dot represents a biological sample. Q,R) sh*Ctrl* and sh*Stk24*‐transfected AKT1 T21 KI CT26 (Q) or LLC (R) cells were subcutaneously transplanted into indicated mice. Each dot represents a biological sample. Results represent at least two independent experiments and are presented as mean ± SEM. ns, no significant difference. ^*^ *p* <0.05, ^**^ *p* <0.01, ^***^ *p* <0.001, ^****^ *p* <0.0001. P values of tumor growth curves were calculated by two‐way ANOVA in (M,N,Q,R). P values in (H,O,P) and P values of tumor weight in (M,N,Q,R) were calculated by unpaired Student's *t*‐tests. See also Figures S5 and S6 (Supporting Information).

The expression of PD‐L1 in tumor cells is mainly regulated by IFN‐γ/STAT1 signal transduction.^[^ ^18^ ^]^ Additionally, the PI3K‐AKT signaling pathway also contributes to IFN‐γ‐induced PD‐L1 expression in tumor cells.^[^ ^19^ ^]^ To elucidate the downstream signaling pathways underlying STK24‐mediated regulation of tumor PD‐L1, a series of key signaling protein candidates were analyzed by western blotting. We found that STK24 deficiency in tumor cells significantly impaired the phosphorylation of AKT, but not STAT1 and STAT3 when cells were exposed to IFN‐γ (**Figure** **5**A–D). Similar signaling phenotypes were also observed in STK24 siRNA‐silenced cells, including CT26, LLC, MC38, and KPC cells (Figure S5A–D, Supporting Information). Consistently, the enrichment of PI3K‐AKT signaling pathway was obtained from the KEGG analysis of LLC cells (Figure S5E, Supporting Information). To verify if STK24 mediates tumor PD‐L1 expression through an AKT activation‐dependent manner, we employed the AKT activation‐specific inhibitor MK2206 to treat *Ctrl* and *Stk24* KO cells prior to IFN‐γ induction. Pre‐treatment with MK2206 significantly inhibited the expression of PD‐L1 in *Ctrl* CT26 or LLC cells (Figure S5F,G, Supporting Information), consistent with previous studies demonstrating the inhibitory impact of MK2206 on PD‐L1 expression.^[^ ^19b^ ^]^ In addition, MK2206 treatment effectively abrogated IFN‐γ‐induced PD‐L1 expression in CT26 or LLC *Ctrl* cells to a level comparable to the *Stk24* KO mutants (Figure S5F,G, Supporting Information), indicating that STK24 regulates PD‐L1 expression in an AKT activation‐dependent manner.

To determine whether STK24 regulates PD‐L1 expression depending on its kinase activity, we introduced a kinase‐dead mutant of STK24 (STK24‐KR, K53R) and wild‐type STK24 into STK24 knockout CT26 or LLC cells. The transfected cells were treated with IFN‐γ and then subjected to flow cytometry analysis. As shown in Figure S5H,I (Supporting Information), wild‐type SKT24, but not the STK24 kinase‐dead mutant, significantly augmented PD‐L1 expression. Consistently, IFN‐γ induced the phosphorylation of AKT was notably elevated by the overexpression of STK24 but not STK24‐KR (Figure S5J,K, Supporting Information). The above results indicate that STK24 kinase activity is critical in modulating the phosphorylation of AKT and PD‐L1 expression.

To reveal how STK24 regulates AKT activation, we carried out co‐immunoprecipitation (CO‐IP) assay to identify STK24‐interacting proteins in HEK293T cells. As shown in Figure 5E, STK24 specifically bound to AKT and the positive control TAK1,^[^ ^8^ ^]^ but not PI3K‐P85 or PI3K‐P55. Subsequent CO‐IP analysis with AKT1, AKT2, and AKT3 in HEK293T cells showed that STK24 interacts with AKT1 but not AKT3. A weak interaction between STK24 with AKT2 was also observed (Figure S5L, Supporting Information). A pull‐down assay further showed that STK24 directly interacted with AKT1 (Figure 5F). We further confirmed physical interaction between STK24 and AKT by immunoprecipitation of endogenous STK24 proteins in MC38 cells and the interaction of the two proteins was augmented by IFN‐γ stimulation (Figure 5G). Consistent with the earlier observations obtained from AKT inhibitor MK2206 (Figure S5F,G, Supporting Information), STK24 silencing failed to inhibit IFN‐γ‐induced PD‐L1 expression in AKT1 knockout cells (Figure S5M,N, Supporting Information; Figure 5H), further confirming that STK24 regulates PD‐L1 expression through AKT1. Domain mapping revealed that the N‐terminus kinase domain (aa1‐313) of STK24^[^ ^20^ ^]^ and the pleckstrin homology (PH) domain (aa1‐160) of AKT1 are indispensable for the interaction (Figure S5O,P, Supporting Information).

We next tested whether AKT1 is a substrate of STK24 kinase activity. Employing purified recombinant proteins, we performed in vitro kinase assays involving EGFP, wild‐type STK24 and the STK24‐KR mutant to phosphorylate EGFP, AKT1‐WT, and the AKT1 S473A/T308A mutant, as indicated in Figure 5I. The reactions were subjected to phos‐tag gel‐mediated western blotting to visualize phosphorylation‐induced protein shift. The results demonstrated that wild‐type STK24 was able to render phosphorylation‐induced protein shift of AKT1, but not STK24‐KR or EGFP. Additionally, the phosphorylation‐induced protein shift of AKT1 was completely abrogated upon calf intestinal alkaline phosphatase (CIAP) treatment, suggesting the phosphorylation‐specific nature of the migration shift of AKT1.

To our surprise, a strong AKT phosphorylation could still be detected when canonical AKT1 phosphorylation sites Thr308 and Ser473 were mutated to alanine (Figure 5I). This result indicates that STK24 phosphorylation of AKT1 can occur at amino acid residue(s) other than Ser473 or Thr308. To identify STK24‐specific phosphorylation site(s) in AKT1, we conducted mass spectrometry analysis on the products of the in vitro kinase assay, revealing three potential target sites: Thr21, Thr82, or Thr291 (Figure S6A, Supporting Information). Subsequently, we generated a series of AKT1 mutants by substituting the three threonine residues to alanine, individually or in combination and tested their phosphorylation in vitro with the STK24 kinase. As shown (Figure 5J–K), the T21/T82 double mutation completely abolished STK24‐mediated phosphorylation, establishing T21 and T82 sites as the specific phosphorylation targets for STK24 on AKT1.

Next, we investigated the functional significance of phosphorylation on T21 and T82 in AKT1 activation. We expressed wild‐type AKT1, T21A, and T82A mutants in AKT1 KO HEK293T (Figure S6B, Supporting Information) or AKT1 KO NCI‐H1299 cells (Figure S6C, Supporting Information). The results show that the T21A mutant but not the T82A mutant, displayed attenuated AKT1 activation. The phosphorylation site of AKT Thr21‐mediated by STK24 was also observed via mass spectrometry in HEK293T cells (Figure S6D, Supporting Information). We then generated a T21 phospho‐specific antibody (pT21‐AKT1) and confirmed its specificity in an ectopic expression setting (Figure S6E, Supporting Information). Using this antibody, we found that STK24 loss in CT26 and LLC cells significantly inhibited IFN‐γ‐induced phosphorylation of AKT at Thr21 (Figure 5L). Furthermore, AKT1 T21D (mimicking AKT1 T21 phosphorylation) almost rescued the impaired IFN‐γ induced PD‐L1 expression in *Stk24* KO CT26 and LLC cells (Figure S6F,G, Supporting Information). Next, we generated T21A knock‐in mutants in CT26 and LLC cells (Figure S6H, Supporting Information) and found that PD‐L1 expression was significantly suppressed in the AKT1 T21A knock‐in cells (KI) (Figure S6I,J, Supporting Information). Moreover, STK24 silencing (Figure S6K,L, Supporting Information) or overexpression (Figure S6M,N, Supporting Information) did not disrupt PD‐L1 expression in AKT1 T21A knock‐in CT26 and LLC cells, further demonstrating that STK24 regulates PD‐L1 expression via phosphorylating AKT1 at the T21 residue.

To investigate the impact of the AKT T21A mutation on tumor cell proliferation and tumor immunity, we measured in vitro cell proliferation in AKT1 T21A knock‐in CT26 and LLC cells. As shown in Figure S6O (Supporting Information), the knock‐in mutant exhibited no detectable difference in cell proliferation compared to the wild‐type (WT) controls. However, in immune‐competent mice, the AKT T21A mutation in CT26 and LLC cells dramatically reduced tumor formation (Figure 5M,N). Flow cytometry analysis revealed that the T21A knock‐in enhanced the infiltration of activated CD8^+^ T cells into tumor tissues of CT26 tumor‐bearing mice (Figure 5O) and activated NK cells and CD8^+^ T cells into tumors tissues of LLC tumor‐bearing mice (Figure 5P).

To verify whether the immunoregulatory role of STK24 in tumors is directly attributable to AKT1 T21phosphorylation, we depleted STK24 in T21A KI CT26 or LLC cells and subsequently inoculated these cells into immune‐competent mice. The knock‐down of STK24 in T21A KI CT26 or LLC cells no longer affected tumor growth and tumor weight (Figure 5Q,R). Similarly, overexpression of STK24 had no effect on the tumor growth in T21A KI CT26 cells (Figure S6P, Supporting Information), strongly supporting the notion that STK24 promotes tumor immune evasion through phosphorylation of AKT1 at T21.

### 2.6 STK24 Downregulation Boosts the Efficacy of the Anti‐PD‐1 Blockade Immunotherapy

**Figure 6:**
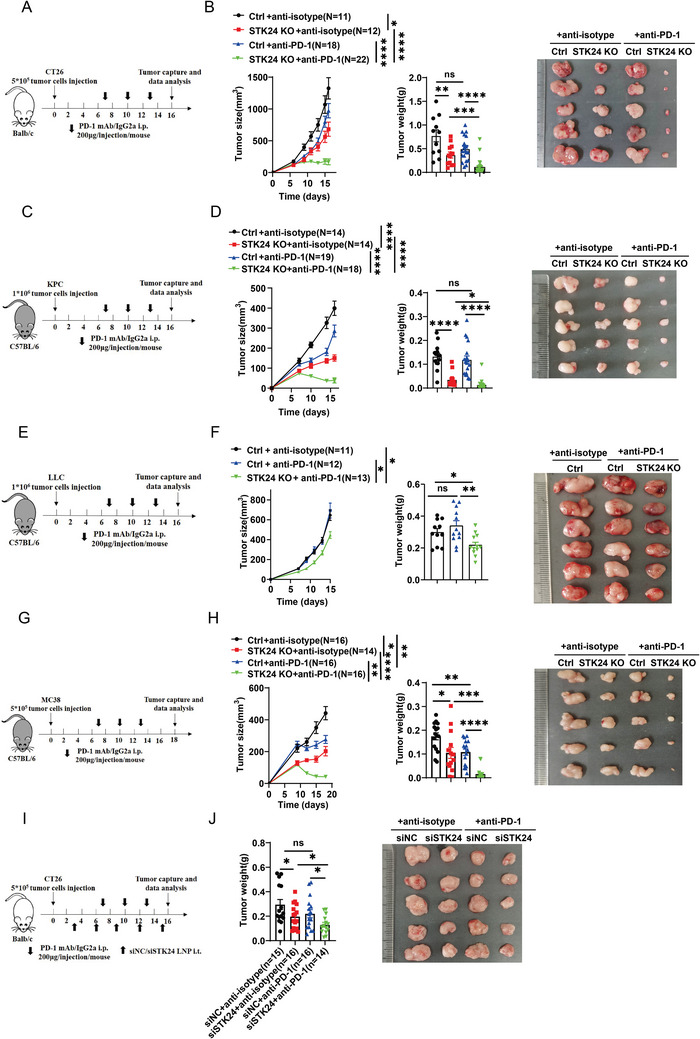
STK24 downregulation overcomes tumor resistance to anti‐PD‐1 blockade immunotherapy. A,B) BALB/c mice were implanted with 5×10^5^ *Ctrl* or *Stk24* KO CT26 cells, and then intraperitoneally injected with the anti‐PD‐1 mAb or the anti‐IgG isotype control (IgG2ɑ). A) Schematic diagram of the treatment plan. B) Tumor growth curves (left), tumor weight (middle), and representative tumor images (right) were shown. C‐H) C57BL/6 mice were implanted with 1 × 10^6^ *Stk24* KO KPC cells (C,D), 1×10^6^ *Stk24* KO LLC cells (E,F), 5 × 10^5^ *Stk24* KO MC38 cells (G,H) and corresponding *Ctrl* cells, and then intraperitoneally injected with the anti‐PD‐1 mAb or the anti‐IgG isotype control (IgG2ɑ). I,J) BALB/c mice were implanted with 5 × 10^5^ CT26 cells and then injected intratumorally with 10 µg si*Stk24* or control LNP, following intraperitoneal injection of the anti‐PD‐1 mAb or the anti‐IgG isotype control (IgG2ɑ). G) Schematic diagram of the treatment plan. H) tumor weight (left) and representative tumor images (right) were shown. Results represent at least two independent experiments and are presented as mean ± SEM. Each dot represents a biological sample. ns, no significant difference. ^*^ *p* <0.05, ^**^ *p* <0.01, ^***^ *p* <0.001, ^****^ *p* <0.0001. P values of tumor growth curves were calculated by two‐way ANOVA in (B,D,F,H). P values of tumor weight were calculated by unpaired Student's *t*‐tests in (B,D,F,H,J). See also Figure S7 (Supporting Information).

Anti‐PD‐1 immunotherapy has been successful in a number of malignancies. Nevertheless, certain tumor types, such as colon, lung cancer, and pancreatic tumor, remain characterized by poor responsiveness to anti‐PD‐1 immunotherapy.^[^ ^21^ ^]^ To evaluate whether STK24 deficiency synergizes with immune checkpoint blockade therapy, the anti‐PD‐1 mAb or the anti‐IgG mAb was *i.p*. injected into the immune‐competent mice inoculated with STK24 knockout tumor cells (*Stk24* KO) or the wild‐type control (*Ctrl*) (**Figure** **6**A–F). While CT26, LLC and KPC cells, showed resistance to anti‐PD‐1 immunotherapy,^[^ ^22^ ^]^ loss of STK24 overcomes the intrinsic resistance to the anti‐PD‐1 blockade immunotherapy. What is more, STK24 deficiency also exerted a synergistic effect with the anti‐PD‐1 antibody blockade immunotherapy in MC38 cells (Figure 6G,H), which was responsive to anti‐PD1 immunotherapy.^[^ ^23^ ^]^ Consistent with the previous data (Figure 3A), FACS assay showed much more intratumoral infiltration of cytotoxic CD8^+^T or NK cells in the *Stk24* KO group when compared to the *Ctrl* group. Meanwhile, *Stk24* deficiency in CT26 or MC38 tumor cells combined with the anti‐PD1 therapy strongly boosted the intratumoral infiltration (Figure S7A,B, Supporting Information) and activation of CD8^+^T or NK cells (Figure S7C,D, Supporting Information).

To determine the potential of STK24 inhibition as a strategy for overcoming tumor immune evasion in vivo, we investigated the delivery of exogenous *Stk24* siRNA into tumor tissues to enhance the immunotherapeutic effect. A lipid‐based nanoparticle (LNP) delivery system was used to generate LNP‐si*Stk24* or LNP‐siNC nanoparticles reagents, a non‐viral system capable of delivery nucleic acids and small molecules into cells.^[^ ^24^ ^]^ Subcutaneously inoculated CT26 tumors in BALB/c mice were injected LNPs containing si*Stk24* or control siRNA. As shown in Figure 6I,J, in vivo silencing STK24 by LNPs containing si*Stk24* RNA inhibited CT26 cell tumorigenesis in mice compared to the siNC control group. What's more, the combined therapy involving LNP‐si*Stk24* and anti‐PD‐1 mAb significantly inhibited tumor growth (Figure 6I,J). Collectively, these results provide proof‐of‐principle that in vivo silencing STK24 could serve as a potential therapeutic approach, synergizing with immune checkpoint therapy.

### 2.7 The Expression of STK24 Correlates with Phosphorylation of AKT‐T21 and PD‐L1 Expression but Inversely Correlates with the Immune Active Status in Human Tumor Specimen

**Figure 7:**
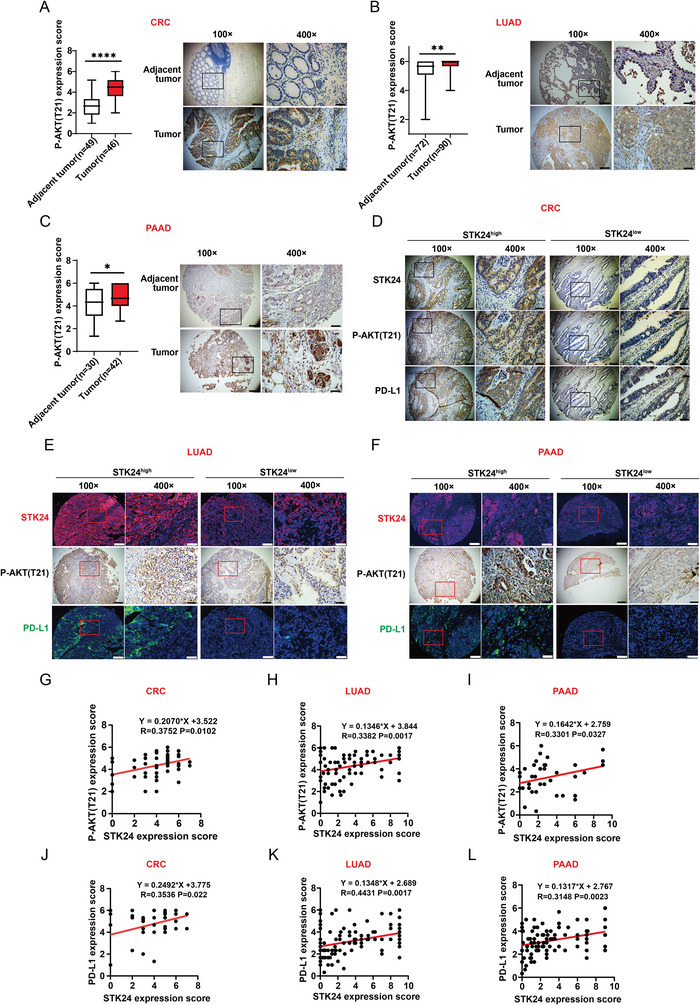
STK24 expression correlates with PD‐L1 expression, AKT1‐T21 phosphorylation and immune cell infiltration in tumor specimens of multiple cancer types. A–C) Immunohistochemical analysis of P‐AKT1 (T21) expression in adjacent‐tumor tissues and tumor tissues from the patients with A) CRC, B) LUAD, or C) PAAD. D–F) Immunohistochemical analysis for STK24 (upper), P‐AKT1(T21) (middle), and PD‐L1 (down) expression in tumor specimens from the patients with D) CRC, E) LUAD, or F) PAAD. 100×, scale bars, 200 µm; 400×, scale bars, 50 µm. G–I) Correlation analysis for STK24 with P‐AKT1 (T21) expression in tumor tissues from the patients with CRC ((G) *n* = 46), LUAD ((H) *n* = 84) or PAAD ((I) *n* = 42). J–L) Correlation analysis for STK24 with PD‐L1 expression in tumor tissues from the patients with CRC ((J) *n* = 46), LUAD ((K) *n* = 96), or PAAD ((L) *n* = 90). Results are presented as mean ± SEM. ^*^ *p* <0.05, ^**^ *p* <0.01, ^****^ *p* <0.0001. P values were calculated by unpaired Student's *t*‐tests in (A–C) and the Pearson correlation test in (G–L). See also Figure S8 (Supporting Information).

To extend our findings to human samples, we assessed protein expression levels of STK24, PD‐L1, and phosphorylated AKT‐T21 by IHC or immunofluorescence (IF) staining of TMA. A higher phosphorylation level of AKT‐T21 was observed in human CRC tumor tissues (**Figure** **7**A), lung cancer tissues (Figure 7B), and pancreatic cancer tissues (Figure 7C) compared to the adjacent tumor tissues. Quantitatively standardized IHC analyses revealed that the expression level of STK24 protein positively correlated with the phosphorylation levels of AKT‐T21 and PD‐L1 expression in these tumors (Figure 7D–L). The positive correlation of gene expression of *STK24* and *CD274(PD‐L1)* was confirmed in LUAD and PAAD patients in the TIMER database (Figure S8A,B, Supporting Information). Moreover, immunofluorescence staining for CD8 and GZMB revealed a significant augmentation of GZMB^+^CD8^+^ T cells in CRC (Figure S8C,D, Supporting Information) or pancreatic cancer patients (Figure S8E,F, Supporting Information) with low expression of STK24.

We next analyzed the correlation between STK24 expression and intratumoral immune cell infiltration based on the TIMER and TISIDB databases. As shown in Figure S8G (Supporting Information), elevated STK24 expression exhibited a concomitant reduction in the infiltration of CD8^+^ T cells or NK cells in tumors of patients with COAD, LUAD, LUSC, READ, and PAAD in TISIDB databases. Consistently, STK24 expression negatively correlated with NK infiltration (Figure S8H, Supporting Information) in tumors of LUSC patients, as well as CD8^+^ T cells infiltration (Figure S8I, Supporting Information) in these four tumors in the TIMER databases. Collectively, these results further indicate that STK24 inhibits anti‐tumor immune response via regulating PD‐L1 expression and AKT Thr21 phosphorylation.

## 3 Discussion

Predictable efficacies and patient stratification markers are unmet clinical needs in cancer immunotherapy. A major portion of treatment failure stems from masked tumor immunity. In this study, we uncovered a regulatory role of STK24 in tumor immunity. Experimental evidence gathered from murine tumor models and patient specimen indicates that STK24 deficiency in tumor cells suppresses tumor growth by orchestrating infiltration of activated CD8^+^ T cells and NK cells. At the molecular level, STK24 phosphorylates AKT1 at Thr21, resulting in the induction of PD‐L1 expression and facilitating tumor immune escape in vivo. Taking advantage of these findings, we obtained positive evidence that STK24 downregulation synergizes with an anti‐PD‐1 immunotherapy in mouse models. Further attesting to our conclusions, we observed an upregulation of STK24 expression in various tumor tissues, correlating with poor survival of patients as well as the intratumoral infiltration of activated CD8^+^ T cells in human cancer tissues.

STK24, belonging to the GCKIII subfamily, is implicated in diverse cellular processes encompassing cell cycle regulation and apoptosis.^[^ ^7^ ^]^ Sequencing analyses of tumor tissues from Chinese patients with small cell lung cancer revealed that altered STK24 transcripts levels as a prevalent occurrence, accounting for 11.5% of all patient samples.^[^ ^25^ ^]^ Database analysis revealed that STK24 expression inversely correlates with overall survival and recurrence‐free survival of NSCLC patients.^[^ ^26^ ^]^ Notably, in our study, increased STK24 expression was found in multiple cancer types such as colorectal, lung, pancreatic, and cholangiocarcinoma cancer, and closely correlated with unfavorable prognosis based on the HPA database and clinical samples.

Our investigation illuminates the multifaceted role of STK24 in tumorigenesis, extending beyond cancer cell autonomy. The attenuation of STK24 expression emerges as a suppressive factor in the growth of tumors including colorectal, lung, and pancreatic cancer cells, particularly in immunocompetent mice. Importantly, STK24 depletion does not affect the proliferation of these tumor cells in vitro. A recent paper suggests that STK24 regulates STAT3/VEGFA signaling pathway and promotes cell cycle in human non‐small cell lung cancer cell lines.^[^ ^12^ ^]^ However, in our experimentation with A549 cells, neither the suppression nor overexpression of STK24 yielded detectable effect on cell cycle dynamics, proliferation, or STAT3 expression (Data not shown). Intriguingly, we employed the identical gRNA sequence as delineated in the aforementioned study to derive *STK24*‐KO A549 cells.^[^ ^12^ ^]^ We did not observe distinguishable differences in cell proliferation or STAT3 expression between *Ctrl* and *STK24* KO A549 cells (data not shown). The inconsistency between our findings and the aforementioned study may potentially be attributed to variations in cell clones or discrepancies in cell passage number. Moreover, STK24 essentiality in cell proliferation has been systematically determined by genome‐wide gRNA dropout screens and is found to be generally dispensable for sustained cell growth in numerous cancer cell lines.^[^ ^27^ ^]^ Hsu et al. reported that STK24 knockout in gastric cancer cells facilitated orthotopic gastric cancer tumorigenesis by promoting the expansion of MDSCs.^[^ ^13^ ^]^ However, in mice bearing colorectal, lung, or pancreatic tumors, we observed primarily the enhanced proportion and amplitude of cytotoxic CD8^+^T cells and NK cells in tumor tissues. Consistently, the levels of STK24 are inversely associated with the number of cytotoxic CD8^+^ T cells in human tumor tissues.

In the tumor microenvironment, the PD‐1/PD‐L1 axis is hijacked by tumor cells to evade immune surveillance.^[^ ^28^ ^]^ Inflammatory signaling pathways such as IFN‐γ, IFN‐α, IL‐6, and oncogenic signaling pathways promote tumor immune escape by up‐regulating PD‐L1 expression level in tumor cells. Various oncogenic pathways such as EGFR, PI3K, AKT, JAK, MYC and ALK have been recently implicated in tumor immunology aside from their cell‐autonomous tumorgenicity roles: affecting the function of CD8^+^ T cells, NK cells, macrophages, and myeloid‐derived suppressor cells (MDSCs) in TME via regulating PD‐L1 expression, ultimately contributes to tumor immune escape and drug resistance.^[^ ^29^ ^]^ Here, we showed that the absence of STK24 in tumor cells results in a suppression of PD‐L1 expression. Importantly, PD‐L1 deficiency in tumor cells abrogated the tumor growth delay in STK24 knockdown tumor cells. We further identified SKT24 promotes PD‐L1 expression via AKT activation.

AKT hyperactivation is associated with many pathophysiological conditions, including human cancers.^[^ ^30^ ^]^ The phosphorylation of two key residues on AKT, including T308 regulated by the phosphoinositide‐dependent protein kinase 1 (PDK1) and S473 regulated by the mammalian target of rapamycin complex 2 (mTORC2), is indispensable for maximal activation of the kinase.^[^ ^31^ ^]^ We here demonstrated that STK24 modulates IFN‐γ induced AKT1 activation via phosphorylating AKT1 at Thr21, a previously unrecognized phosphorylation site. The evolutionary conservation of Thr21 in AKT1 underscores its functional significance (Figure S8J, Supporting Information). AKT T21A knock‐in not only abolished PD‐L1 downregulation but also compromised tumor growth in murine syngeneic models upon STK24 silencing. Moreover, IHC staining of the human TMA showed STK24 expression is correlated with PD‐L1 expression and phosphorylation of AKT at T21 residue. Meanwhile, an elevated level of phosphorylation at AKT1‐T21 was observed in human CRC, lung cancer and pancreatic cancer tissues. AKT is recruited to the plasma membrane by phosphatidylinositol 3,4,5‐triphosphate (PIP3) through its PH domain, which is considered a prerequisite for AKT full activation.^[^ ^32^ ^]^ Arg23 has been reported to play a critical role in PIP3 binding^[^ ^33^ ^]^ and Thr21 locates in a region of the PH domain. Structural illustration showed that the Arg23 interacts with Thr21 since their distances is <5Å, indicating that Thr21 site might impact the complete activation of AKT (Figure S8K, Supporting Information). Thr21 mutation of AKT1 has also been identified in human breast, lung, and ovarian cancers, as evidenced by data from the COSMIC and Bioportal databases, thus highlighting the potential clinical significance of AKT1 T21 phosphorylation in these cancers.

AKT is an important regulator of cellular processes, such as cell signaling, survival, and proliferation.^[^ ^34^ ^]^ In our present study, STK24 emerges as a notable facilitator of AKT activation, without appreciably perturbing tumor cell proliferation. A similar phenotype has been observed in interleukin (IL)−27 receptor WSX1‐mediated tumor growth regulation. WSX1 intervenes in hepatocellular carcinoma (HCC) tumorigenesis by blocking the PI3Kδ/AKT/GSK3β/PD‐L1 pathway, without affecting the proliferation of HCC cells in vitro.^[^ ^35^ ^]^ A parallel observation reported that the P29S mutation of RAC1, RHO family small GTPase, which activates AKT without altering cell proliferation.^[^ ^36^ ^]^ These observations, combined with our results, seem to suggest that AKT activity can be modularized and/or compartmentalized depending on cell type or the nature of stimuli. It is also possible that AKT phosphorylation at Thr21 residue may induce changes in kinase specificity toward different substrates, elevating transcriptional PD‐L1 expression level yet maintaining normal cell proliferation. The mechanism underlying this phenotype remains to be explored.

Therapeutic targeting of the immune checkpoints by PD‐1 and PD‐L1 has been approved for multiple cancer types with considerable effect. However, the response rate to PD‐L1/PD‐1 blockade remains modest, with <40% of patients exhibiting favorable outcomes. Substantial efforts are being channeled toward identifying novel targets to enhance the efficacy of immunotherapy through combination treatments.^[^ ^37^ ^]^ Our investigation demonstrates that the abrogation of STK24, achieved through both genetic deficiency and LNP‐mediated siRNA silencing, significantly improves the efficacy of anti‐PD‐1 blockade immunotherapy in mouse models. Intratumor injection of siSTK24 may have a profound role in immune cells. However, few studies reported the function of STK24 in immune cells including regulating neutrophil degranulation^[^ ^38^ ^]^ and NLRP3 inflammasome activation in macrophage.^[^ ^9^ ^]^ The immune cell‐specific effects of STK24 in tumorigeneses need further delineation in the future.

Given that STK24 facilitates tumor immune evasion through its kinase activity, the prospect of employing small molecules specifically designed to target STK24 enzyme activity is a plausible strategy for combination therapy with an immune‐checkpoint blockade. While AKT1 and AKT2 manifest widespread expression across diverse cell types.^[^ ^36^ ^]^ AKT3 is limitedly expressed in the nervous system based on the analysis of the HPA database (data not shown). The dominant AKT isoform in various tumor tissues is not well established. The Thr21is conserved across AKT1 and AKT2, but the corresponding position in AKT3 does not contain a serine or threonine residue (Figure S8L, Supporting Information). It should be more cautious when employ a STK24‐targeted strategy in tumor immunotherapy.

In conclusion, our study revealed the pivotal role of STK24 in orchestrating tumor immune evasion responses by phosphorylating AKT and promoting PD‐L1 expression (Figure S8M, Supporting Information). STK24 inhibition effectively overcomes tumor intrinsic resistance to anti‐PD1 therapy. Given the up‐regulation of STK24 gene expression in various tumor tissues and its correlation with poor survival, STK24 could be a promising target for the development of more effective immunotherapeutic interventions.

## 4 Experimental Section

### Mice

*Stk24* ^h/+^ mice were crossed to obtain wild‐type (WT) or *Stk24* ^h/h^ (homozygous deletion) littermates. Mice were genotyped by PCR analysis of DNA isolated from the tail using the primers: 5′‐AAAGCGGTGGGGAAATTAGAAAA‐3′; 5′‐CTCTGTA TAGCCCTGGCTGCATACAA‐3′; 5′‐GGCACCCACGACCTGGCTTA‐3′. C57BL/6 and BALB/C mice were purchased from the Shanghai SLAC Laboratory. NSG mice were purchased from the Shanghai Model Organisms Center. The mice at the age of 6–8 weeks were employed in experiments. All the mice were kept in specific pathogen‐free conditions and the animal experiments were performed with approval from the Institutional Animal Care and Scientific Investigation Board of Zhejiang University (Authorized N.O. ZJU2015‐040‐01).

### In Vivo Treatments

For the immunodeficient mouse model, *Stk24* KO CT26 cells (5 × 10^5^) or LLC cells (1 × 10^6^) and their control cells were injected subcutaneously into NSG mice in a volume of 100 µL medium. For the immune‐competent mouse model, *Stk24* KO, or *Ctrl* CT26 cells (5 × 10^5^) were injected subcutaneously into Balb/c mice and *Stk24* KO MC38 cells (5 × 10^5^), LLC cells (1 × 10^6^) or KPC cells (1 × 10^6^) and their control cells were injected subcutaneously into C57BL/6 mice. Measured tumors in the long and short dimensions using digital calipers, and recorded tumor volumes using the formula: V = W × L^2^ × 0.5, where W represents the largest tumor diameter in centimeters and L represents the next largest tumor diameter. Tumor tissues were harvested for weight measurement and further analyses. For the in vivo depletion of CD8^+^ T cells experiments, *Ctrl* and *Stk24* KO CT26 tumor‐bearing mice were intraperitoneally injected with mouse‐CD8α mAb (BP0117) or IgG isotype control (BP0090) 1 day before tumor inoculation and then once every three days to ensure sustained depletion of CD8^+^ T cells. For in vivo immune checkpoint blockade experiments, *Stk24* KO CT26, MC38, LLC, or KPC tumor‐bearing mice were intraperitoneally injected with mouse‐PD‐1 mAb (BE0146) or IgG isotype control (BE0089). PD‐1 mAb was given on day 7 after tumor cell inoculation and every 3 days for the duration of the study.

### Urethane‐Induced Mouse Primary Lung Cancer Model

*Stk24* ^h/h^ and wild‐type mice (WT) were kept in the SPF sterile environment at the Animal Experiment Center, Zhejiang University School of Medicine. Urethane was dissolved in a sodium chloride solution. 6 to 7‐week‐old *Stk24* ^h/h^ and WT mice were intraperitoneally injected with 1 mg g^−1^ of urethane solution weekly for 10 weeks as previously described^[^ ^39^ ^]^ and sacrificed mice after 30 weeks from the first urethane injection.

### In Vitro Kinase Assay

The relevant plasmids of fusion proteins were transfected in HEK293T cells, and the cell extracts were lysed by using lysis buffer (50 mm Tris, pH 7.4, 150 mm NaCl, and 0.5% (vol/vol) Nonidet P‐40, 1 mm EDTA) supplemented with a protease inhibitor cocktail (Roche). Then the proteins were purified according to protocols of the Ni‐NTA Column purchased from Sangon Biotech (Shanghai, China) (#C600791). The purified proteins were re‐suspended in 40 µL of 1×kinase buffer (Cell Signaling, #9802) supplemented with 200 mm ATP (Cell Signaling, #9804), and then the reactions were carried out at 30 °C for 60 min. The reactions were stopped with 2×SDS loading buffer, and then the samples were subjected to SDS‐PAGE and immunoblot analysis using indicated antibodies.

### Immunohistochemical and Immunofluorescence

Immunohistochemistry staining of the tissue microarray (TMA) of colorectal cancer was performed by Servicebio. Lung adenocarcinoma and pancreatic carcinoma TMA were purchased from Shanghai outdo biotech co., LTD, and the immunohistochemistry staining was performed by Recordbio. The staining extent score was performed as previously described.^[^ ^40^ ^]^ The score was on a scale of 0–3, corresponding to the percentage of immunoreactive tumor cells (0−10%, 11−25%, 26−75%, and 76−100%, respectively) and the staining intensity (negative, score = 0; weak, score = 1; strong, score = 2; very strong, score = 3). A score ranging from 0–3 was calculated by adding the staining extent score with the intensity score, resulting in a low (< median) level or a high (≥ median) level value for each specimen. Immunofluorescence staining of the TMA was performed by Recordbio. The scanning analysis was carried out on Core Facilities, Zhejiang University School of Medicine. The number of CD8^+^, GZMB^+^, and CD8^+^GZMB^+^ was counted using image J software.

### Statistical Analysis

Statistical analysis was performed using GraphPad Prism 8 software. Differences were considered significant at a P value of <0.05. Unpaired Student's t test was used to calculate the P values for comparisons of tumor numbers, tumor‐infiltrating cells, and relative mRNA expression levels, or quantitative evaluation of immunohistochemical staining. Two‐way ANOVA was used for multiple comparisons in tumor growth. Correlation studies were analyzed using the Pearson correlation factor r. Kaplan–Meier survival analysis was performed with the log‐rank (Mantel–Cox) test.

## Conflict of Interest

The authors declare no conflict of interest.

## Author Contributions

N.W., Y.J., and M.L. contributed equally to this work. N.W., Y.J., M.L., H.W., J.P., Y.T. and Y.X. conducted experiments. N.W., Y.J., M.L. and H.W. performed the statistical analysis. S.X. and X.L. performed mass spectrometry analyses. X.Z. provided the help for LNPs formulation. P.X. provided the help for knock‐in cells generation. X.W. and W.L. designed the study. X.W., W.L. and N.W. drafted the manuscript.

## Supporting information

Supporting Information

## Data Availability

The data that support the findings of this study are available from the corresponding author upon reasonable request.
