# Supplementary material for: Protein Kinase STK24 Promotes Tumor Immune Evasion via the AKT‐PD‐L1 Axis
Source: Adv Sci (Weinh). 2024 Jan 16;11(12):2304342. doi: 10.1002/advs.202304342 (PMC10966517; doi:10.1002/advs.202304342)
Supplement: Supplementary file 1 — Supporting Information [file ADVS-11-2304342-s001.pdf]

ADVANCED SCIENCE
Open Access

Supporting Information

for *Adv. Sci.*, DOI 10.1002/advs.202304342

Protein Kinase STK24 Promotes Tumor Immune Evasion via the AKT-PD-L1 Axis

Ning Wang, Yu Jiang, Mengjie Li, Haofei Wang, Jie Pan, Yang Tang, Shaofang Xie, Yunyang Xu, Xu Li, Xuefei Zhou, Pinglong Xu, Wenlong Lin and Xiaojian Wang**

Protein kinase STK24 promotes tumor immune evasion via the AKT-PD-L1 Axis

Ning Wang[1#], Yu Jiang[2#], Mengjie Li[1#], Haofei Wang[1], Jie Pan[1], Yang Tang[1], Shaofang Xie[3], Yunyang Xu[1], Xu Li[3], Xuefei Zhou[4], Pinglong Xu[5], Wenlong Lin[1*] and Xiaojian Wang[1*]

1. Institute of Immunology and Bone Marrow Transplantation Center, The First Affiliated Hospital, School of Medicine, Zhejiang University, Hangzhou 310058, Zhejiang, China.
2. Department of Clinical Laboratory, Second Affiliated Hospital of Zhejiang University, School of Medicine, Hangzhou 310058, Zhejiang, China.
3. Westlake Laboratory of Life Sciences and Biomedicine, School of Life Sciences, Westlake University, Hangzhou 310024, Zhejiang, China.
4. Department of Pharmacology, School of Medicine, Zhejiang University, Hangzhou 310058, Zhejiang, China
5. Life Sciences Institute, Zhejiang University, Hangzhou 310058, Zhejiang, China

1. Supplementary methods

2. Table S1. List of antibody information

3. Supplementary Figures and legends

Supplementary Figure 1, related to Figure 1;

Supplementary Figure 2, related to Figure 2;

Supplementary Figure 3, related to Figure 3;

Supplementary Figure 4, related to Figure 4;

Supplementary Figure 5, related to Figure 5;

Supplementary Figure 6, related to Figure 5;

Supplementary Figure 7, related to Figure 6

Supplementary Figure 8, related to Figure 7

Supplementary methods

Antibodies and plasmids

Antibodies for phospho-AKT(Ser473) (#4060S), phospho-STAT3(Tyr705) (#9145S), phospho-STAT1(Tyr701) (#9167S), AKT (#4691S) and PD-L1 (#13684S) were purchased from Cell Signaling Technology (Cell Signaling Technology, Beverly, MA). The antibody for STK24 (#ab51137) was purchased from Abcam. Antibody for Flag (#AE063) was purchased from Abclonal. Antibodies for Myc (#R1208-1) and HA(#R0906-1) were purchased from HUABIO. Brilliant Violet 605 anti-mouse CD45 (#103140), PE anti-mouse CD4 (#100408), Brilliant Violet 650™ anti-mouse CD8α (#100742), APC anti-mouse TCR β chain (#109212), FITC anti-human/mouse Granzyme B Recombinant (#372206), Alexa Fluor® 647 anti-human/mouse Granzyme B (#515406), PE/Cyanine7 anti-mouse IFN-γ (#505826), Pacific Blue anti-mouse/human CD11b (#101224), PE anti-mouse F4/80 (#123110), APC anti-mouse Ly-6C (#128015), PE/Cyanine7 anti-mouse CD11c (#117318), APC/Cyanine7 anti-mouse I-A/I-E (#107628), PE/Cyanine7 anti-mouse CD274 (B7-H1, PD-L1) (#124313) and PE/Cyanine7 anti-human CD274 (B7-H1, PD-L1) (#329717) were purchased from Biolegend. Mouse IFN gamma (#CM41) and human IFN gamma (#C014) recombinant proteins were purchased from Novoprotein. *In viv*o mAb anti-mouse PD-1 (#BE0146) and *in vivo* mAb antanti-mouse8α (#BP0117) were purchased from Bio X Cell. Expression plasmids encoding Flag-STK24, Flag-STK24-K53R, Flag-STK24-N domain, Flag-STK24-C domain, and Myc-STK24 have been described previously[1]. Flag-AKT and Flag-AKT deletion mutants were subcloned into the pcDNA3.1-Flag-His vector. HA-AKT and site-directed mutants were subcloned into the pcDNA3.1-HA-His vector.

Human Subjects

We obtained a tissue microarray (TMA) of colorectal cancer tissues and adjacent non-tumor tissues from the First Affiliated Hospital and Sir Run Shaw Hospital of Zhejiang University (2005DKA21300). The tissue microarray (TMA) of lung adenocarcinoma (SHYJS-CP-1904008) and pancreatic adenocarcinoma (SHYJS-CP-1901009) were

obtained from the Shanghai Outdo Biotech Company.

Cell culture, plasmid transfection, and siRNA silencing.

CT26, MC38, LLC, KPC, NCI-H1299, HCT116, and A549 cell lines were obtained from American Type Culture Collection. Lipofectamine 3000 (Invitrogen) or polyethylenimine (Polysciences) transfection reagents were used for the plasmid transfections. Scramble siRNA and STK24-targeted siRNA were transfected in cells, using INTERFERin@ according to the manufacturer's protocol. The following siRNA oligonucleotide sequences were used: human siRNA:5′-GCUCCGCACUAGAUCUAUUTT -3′, mouse siRNA:5′-AAGGCAUMGACAAUCGGACUCAGAA -3′.

Generation and validation of STK24-KO, AKT1-KO, PD-L1-KO, and AKT1 T21A knock-in cells.

STK24, AKT1, and PD-L1 (*CD274*) were knocked out in tumor cell lines using the CRISPR/Cas9 gene-editing system. CRISPR/Cas9 genomic editing for gene deletion was performed as previously described[2]. Guide RNA (gRNA) sequences targeting the STK24 and PD-L1 genomic sequence (hSTK24: 5′-CCATGACGACTCGAGCTCCG -3′ and 5′- CGGAGCTCGAGTCGTCATGG -3′; mStk24 1#: 5′-CAGGTACAAGAGGTGGAAGG -3′ and 5′- CCTTCCACCTCTTGTACCTG -3′; mSTK24 2#: 5′- TGGAGTGGCCGGCCAGCTGA -3′ and 5′-TCAGCTGGCCGGCCACTCCA -3′; mPd-l1 1#: 5′-TCACCACTTCCCGGACAGAG -3′ and 5′- CTCTGTCCGGGAAGTGGTGA -3′;) were used to clone the genes of interest into the plasmid. Plasmids of gRNA targeting the AKT1 genomic sequence (hAKT1: 5′- CTGCTTTGTCATGGAGTACG -3′ and 5′-CGTACTCCATGACAAAGCAG -3′) and AKT1 KO HEK293T cells were provided by Dr. Pinglong Xu. To generate KO cells, these constructs were co-transfected with Cas9-2A-GFP into CT26, MC38, LLC, KPC, NCI-H1299, A549, or HCT116 cells with Lipofectamine 3000 (Invitrogen). 48 hours after transfection, cells with green fluorescence were sorted with a flow cytometer (BD FACSAria II). Clones were identified by sanger sequence, immunoblotting or FASC with corresponding antibodies.

Akt1 gRNA sequences of knock-in (mAkt1, 5′- CAGGGGAATATATTAAAACC -3′ and 5′-GGTTTTAATATATTCCCCTG-3′) were used to clone the genes into the plasmid gRNA. The AKT1 genomic sequence was cloned into the plasmid pMD18 and T21 site was mutated from ACC to GCC. These constructs were transfected with Cas9-2A-GFP and pMD18 into CT26 or LLC cells. Cells with green fluorescence were then sorted with a flow cytometer (BD FACS Aria II) 48 hours after transfection. Clones were identified by digestion/sequencing identification of genomic PCR products.

Cell proliferation assay

Cells were plated at a density of 3000 cells except for HCT116, LLC and A549 cells at a density of 5000 cells per well in 96-well culture plates and starved overnight. The serum stimulation was a time-decreased course, and plates were read at 96 hours. The number of cells could be represented by the different absorbances measured using the SynergyMx M5 (Molecular Devices).

Generation of the AKT1 T21 phosphorylation antibody.

The AKT1 T21 phosphorylation antibody was produced by Jingyun Biotechnology Co., LTD Shanghai based on the human AKT1 protein sequence. The immunopeptide sequence is CGEYIKp(T)WRPRY and the control peptide is CGEYIKTWRPRY.

Generation of mouse shStk24 lentivirus

The targeting sequences (sh*Stk24* #1, 5′- CGAGATAGAGGACATCCAA -3′ and sh*Stk24* #2, 5′- GGATCCTATCTCAAGGATA -3′) were cloned into PLKO.1 vector and lentivirus were packaged as previously described with little modification. Briefly, transfected with 10μg of sh*Stk24* plasmids together with 10μg of psPAX2 and 5μg of pMD2G into HEK293T cells.10 hours after transfection, changed the media to fresh DMEM containing 10% FBS. Collected and centrifuged the supernatant media after 48 hours. The supernatant was filtered through a 0.45μm membrane and stored at -80 °C.

Flow cytometry analysis

Tumors were collected after mice sacrifice, weighed, mechanically minced, and incubated in DNase I (100 μg/ml, Solarbio) and collagenase IV (1 mg/ml, Sigma) for 60 min at 37 °C. The dissociated cells were filtered with a 70-μm cell strainer. After

blocking with CD16/CD32 (40477) antibody and getting rid of dead cells with Zombie Aqua Fixable Viability Kit (77143), cells were stained with indicated surface antibodies for 20 min on ice. Intracellular antibodies were added after fixation and permeabilization according to the manufacturer's instructions. Stained cells were analyzed by BD Fortessa. Data were further analyzed by Flow Jo 10.0 software.

Pull-down assay

The fusion protein of Flag-STK24-His, HA-EGFP-His, and HA-AKT-His was expressed in HEK293T cells and purified according to standard protocols of the Ni-NTA Column purchased from Sangon Biotech (Shanghai, China) (#C600791). For the HA pull-down assay, approximately 1μg of HA-fusion proteins were mixed with 20μl of pre-cleared anti-HA beads in 500μl of reaction buffer, subsequently, 1μg of Flag-STK24-His protein was added into the mixture and the mixture was incubated at 4°C for 4 hours with gentle rotation. The precipitates were extensively washed three times, and the samples were subjected to SDS-PAGE and immunoblot analysis using indicated antibodies.

Immunoprecipitation

The relevant plasmids were transfected in HEK293T cells as indicated groups and cell extracts were lysed by using lysis buffer (50 mM Tris, pH 7.4, 150 mM NaCl, and 0.5% (vol/vol) Nonidet P-40, 1 mM EDTA) supplemented with a protease inhibitor cocktail (Roche). Next, lysates were incubated with the anti-Flag (M2)-agarose or anti-HA beads (MedChemExpress, HY-K0201) for 4 hours at 4°C. The immunoprecipitants were washed three times with the lysis buffer and subjected to further immunoblot analysis.

Ova-specific cytotoxic CD8$^+$T-cell killing assay

CD8$^+$ T cells were isolated from spleen of OT-1 mice using the EasySeq mouse CD8$^+$ T-cell isolation kit (STEMCELL, 19853) according to the manufacturer's protocol. CD8$^+$ T cells were activated with CD3(2μg/ml) and CD28 (2μg/ml) for 48 hours. And Stk24 knockdown MC38-OVA and KPC-OVA cells were first stimulated with mouse recombinant IFN-γ at 10 ng/ml for 12 hours. OVA-specific T cells were then cultured

with the stimulated tumor cells at the E:T ratio of 1:1 or 2:1 in T-cell complete growth medium with mouse recombinant interleukin-2 (10ng/ml) for 48 hours. Live tumor cells were counted by flow cytometry.

RNA isolation and real-time quantitative PCR

Total RNA was isolated using TRIzol (TAKARA, Ostushiga, JAPAN) and cDNA was synthesized with a reverse-transcription kit (TAKARA, Ostushiga, JAPAN). The expression of genes was detected by a LightCycler 480 system (Rocho) with Master SYBR Green (Tsingke Biotechnology Co., Ltd, Beijing, China). The data was calculated by a standard curve method and normalized to the expression of the gene encoding *β-actin* (mouse). The sequence of primers was as follows:

mβ-Actin forward primer:5'-AACAGTCCGCCTAGAAGCAC-3'

mβ-Actin reverse primer:5'- CGTTGACATCCGTAAAGACC-3'

mPd-l1 forward primer:5'- TGCGGACTACAAGCGAATCACG-3'

mPd-l1 reverse primer :5'- CTCAGCTTCTGGATAACCCTCG-3'

LNPs formulation

DLin-MC3-DMA ionizable lipid, DSPC (1,2-distearoyl-sn-glycero-3-phosphocholine), and DMG-PEG (1,2-dimyristoyl-sn-glycero-3-methoxypolyethyleneglycol 2000) were purchased from AVT (Shanghai) Pharmaceutical Tech CO. Ltd., and cholesterol were purchased from Sigma. For LNPs formulation, lipid components (50% Dlin-MC3-DMA, 10% DSPC, 38.5% cholesterol, 1.5% DMG-PEG, in mol%) were dissolved in ethanol, and siRNAs (siSTK24) were dissolved in 50 mM sodium citrate (pH 4.0). LNP-siRNA was prepared by mixing the organic lipid components and the aqueous RNA solution with a microfluidic laminal flow rate of 800 μL/min (aqueous:organic flow rate ratio of 3:1) using a NanoAssemblr Benchtop (Precision Nanosystems, Vancouver, Canada). Then, the mixture was dialyzed for 12 hours at 4 °C against PBS (pH 7.4) using a Slide-A-Lyzer Dialysis Cassette 20 K MWCO (Thermo Fisher Scientific, Waltham, MA). Resultant LNP was concentrated with an Amicon Ultra 30 K MWCO (Merk Millipore, Burlington, MA).

Table S1. List of antibody information

Antibodies	SOURCE	IDENTIFIRS
phospho-AKT(Ser473)	Cell Signaling Technology	#4060S
phospho-AKT(Thr308)	Cell Signaling Technology	#13038S
phospho-AKT(Thr21)	Jingyun (Shanghai) Biotechnology Co. Ltd	
phospho-STAT3(Tyr705)	Cell Signaling Technology	#9145S
phospho-STAT1(Tyr701)	Cell Signaling Technology	#9167S
AKT	Cell Signaling Technology	#4691S
PD-L1	Cell Signaling Technology	#13684S
STK24	Abcam	#ab51137
Flag Tag antibody	Abclonal	#AE063
Myc Tag antibody	HUABIO	#R1208-1
HA Tag antibody	HUABIO	#R0906-1
BV605 anti-mouse CD45	Biolegend	#103140
PE anti-mouse CD4	Biolegend	#100408
Brilliant Violet 650™ anti-mouse CD8α	Biolegend	#100742
APC anti-mouse TCR β chain	Biolegend	#109212
FITC anti-human/mouse Granzyme B	Biolegend	#372206
PE/Cyanine7 anti-mouse IFN-γ	Biolegend	#505826
Pacific Blue anti-mouse/human CD11b	Biolegend	#101224
PE anti-mouse F4/80	Biolegend	#123110
APC anti-mouse Ly-6C	Biolegend	#128015
PE/Cyanine7 anti-mouse CD11c	Biolegend	#117318
APC/Cyanine7 anti-mouse I-A/I-E	Biolegend	#107628
PE/Cyanine7 anti-mouse CD274 (B7-H1, PD-L1)	Biolegend	#124313
PE/Cyanine7 anti-human CD274 (B7-H1,	Biolegend	#329717

PD-L1)		
Mouse IFN gamma recombinant proteins	Novoprotein	#CM41
Human IFN gamma recombinant proteins	Novoprotein	#C014
*In viv*o mAb anti-mouse PD-1	Bio X Cell	#BE0146
in vivo mAb anti-mouse CD8α	Bio X Cell	#BP0117

Supplementary figures and figure legends

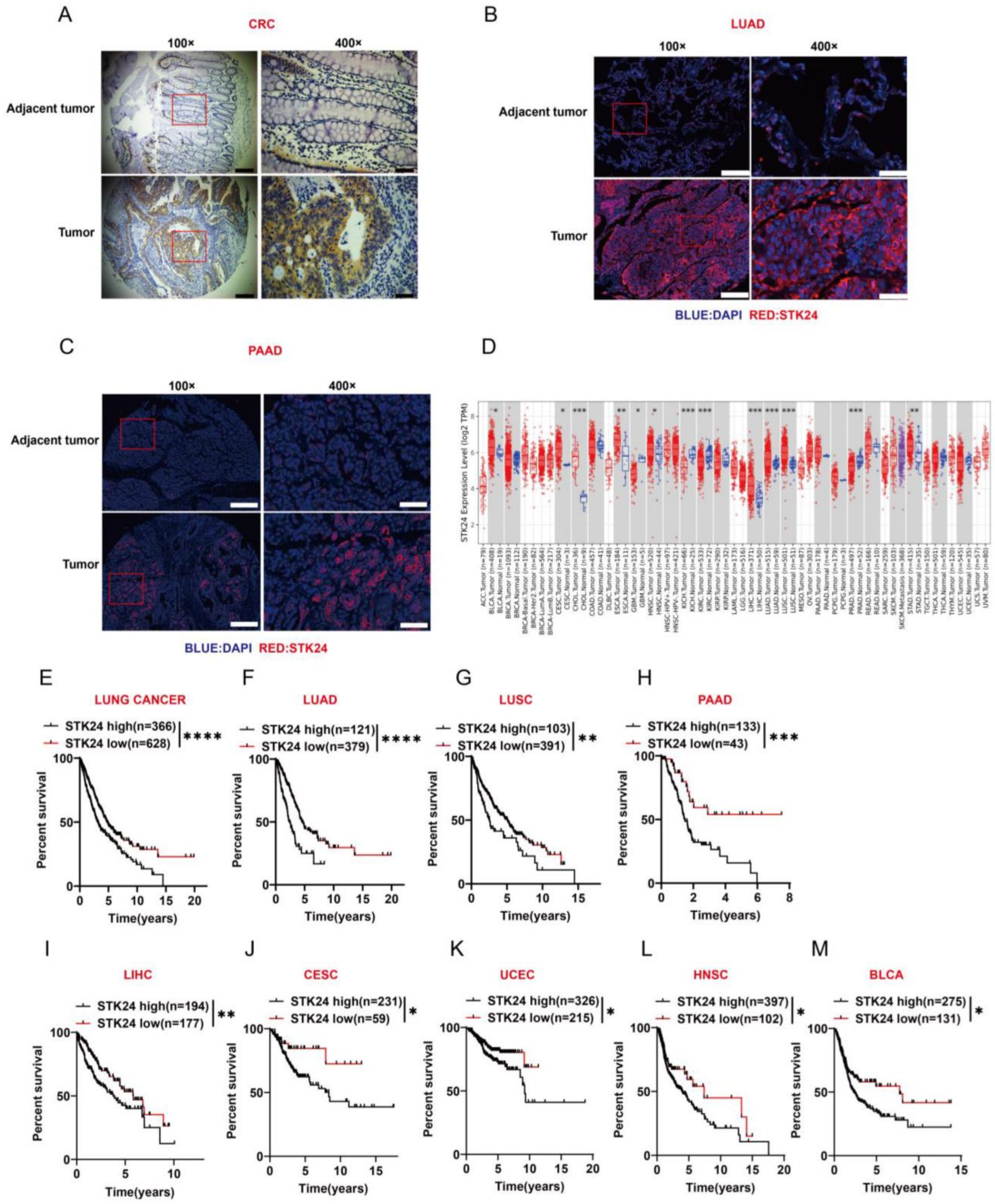

Figure S1. STK24 expression is upregulated in multiple cancer types and negatively correlated with the survival rate.

(A-C) Representative IHC or IF staining of tumor sections from patients with colorectal

cancer (CRC) **(A)**, lung adenocarcinoma (LUAD) **(B)**, or pancreatic adenocarcinoma (PAAD) **(C)** with anti-STK24 antibody. The red boxes are the indicated 400× fields in the 100× sections. 100×, scale bars, 200 μm; 400×, scale bars, 50 μm.

(D) Comparison of STK24 gene expression levels in tumor tissues or normal tissues from patients of various tumors based on the TIMER database.

(E-M) Kaplan-Meier analysis of overall survival in the set of patients with lung cancer **(E)**, LUAD **(F)**, LUSC **(G)**, PAAD **(H)**, liver hepatocellular carcinoma (LIHC) **(I)**, cervical squamous cell carcinoma and endocervical adenocarcinoma (CESC) **(J)**, uterine corpus endometrial carcinoma (UCEC) **(K)**, head and neck squamous cell carcinoma (HNSC) **(L)**, or bladder urothelial carcinoma (BLCA) **(M),** of which the patients were divided into two groups with high STK24 or low STK24 expression in the tumors based on the HPA database.

*P<0.05, **P<0.01, ***P<0.001, ****P<0.0001. P values were calculated by log-rank test.

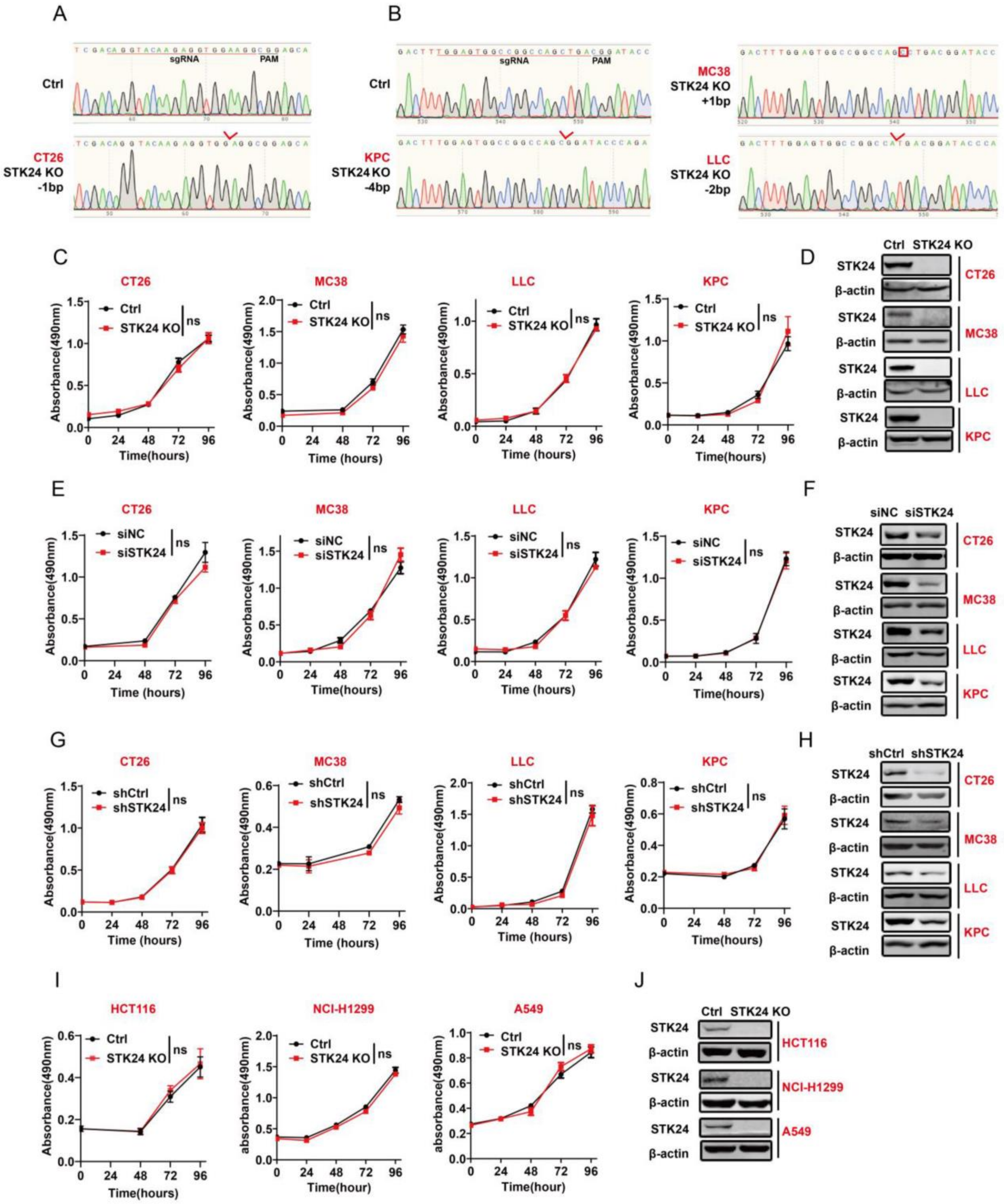
A
B
C
D
E
F
G
H
I
J
sgRNA
PAM
Ctrl
CT26
STK24 KO
-1bp
KPC
STK24 KO
-4bp
MC38
STK24 KO
+1bp
LLC
STK24 KO
-2bp
CT26
MC38
LLC
KPC
Absorbance(490nm)
Time(hours)
Time (hours)
Ctrl
STK24 KO
ns
siNC
siSTK24
shCtrl
shSTK24
STK24
β-actin
HCT116
NCI-H1299
A549
absorbance(490nm)
Time(hour)

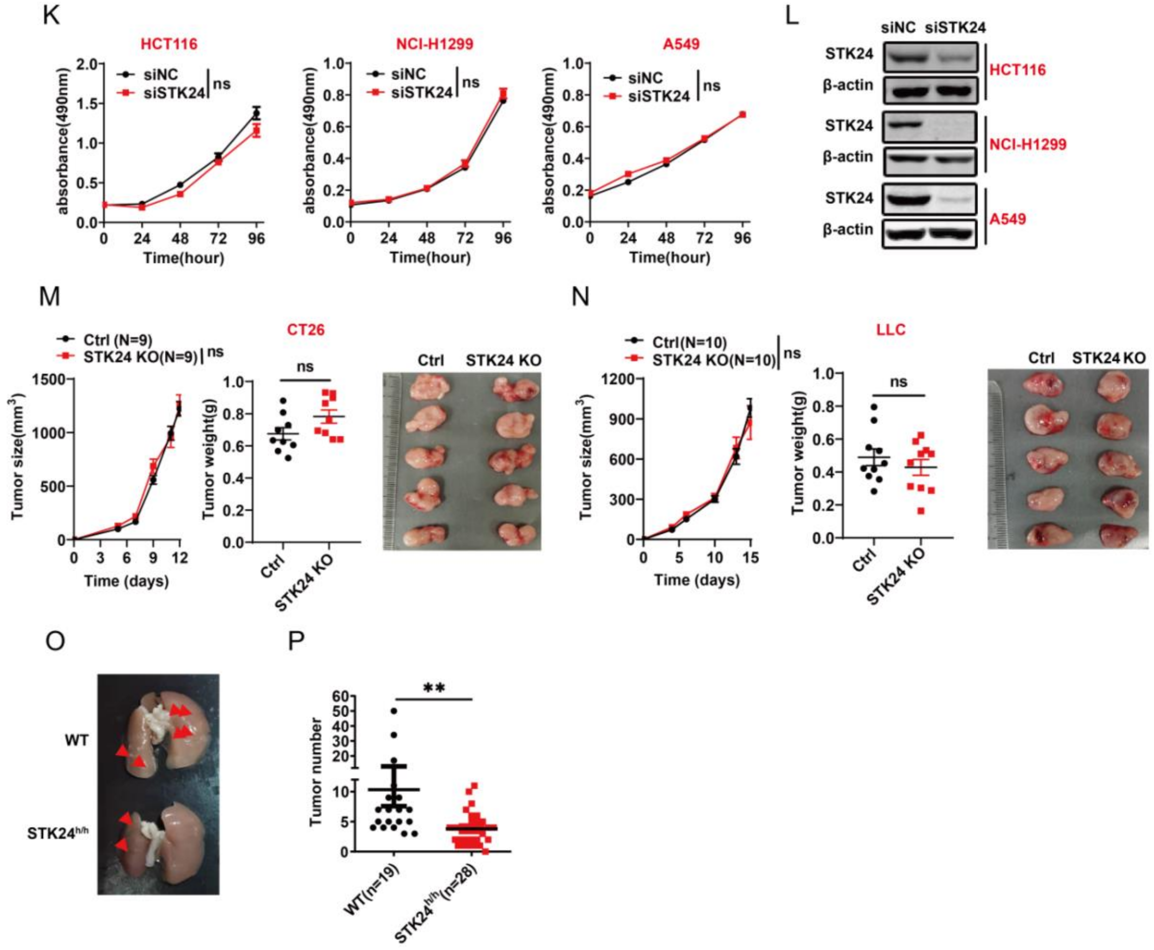

Figure S2. STK24 deficiency does not affect cell growth *in vitro* and *in vivo*.

(A-B) Genomic DNA was extracted from the indicated cells. Sequence analysis of CT26 **(A)**, MC38, KPC and LLC cells **(B)** indicated the knockout of these four cell lines.

(C-D) The proliferation of the *Ctrl* and *Stk24* KO CT26, MC38, LLC, and KPC cells**(C)**. Immunoblot analysis of STK24 expression in above cell lines**(D)**.

(E-F) The proliferation of the CT26, MC38, LLC, and KPC cells transfected with STK24-specific (si*Stk24*) or scrambled (siNC) siRNAs**(E)**. Immunoblot analysis of STK24 expression in above cell lines**(F)**.

(G-H) The proliferation of the CT26, MC38, LLC, and KPC cells transfected with sh*Stk24* or scrambled negative control (sh*Ctrl*) shRNA**(G)**. Immunoblot analysis of STK24 expression in above cell lines**(H)**.

(I-J) The proliferation of the *CTRL* and *STK24 KO* HCT116, NCI-H1299, and A549

cells(**I**). Immunoblot analysis of STK24 expression in above cell lines(**J**).

(K-L) The proliferation of the HCT116, NCI-H1299, and A549 cells transfected with si*STK24* or siNC siRNAs(**K**). Immunoblot analysis of STK24 expression in above cell lines(**L**).

(M-N) *Ctrl* and *Stk24* KO CT26 cells (5×10^5, **M)** or LLC cells (1×10^6, **N)** were subcutaneously transplanted into NSG mice. The figures show tumor growth curves **(left)**, tumor weight **(middle)**, and representative images **(right)**. Each dot represents a biological sample.

(O-P) *Stk24*$^{h/h}$ and wild-type (WT) mice were intraperitoneally injected with urethane dissolved in saline weekly for 10 weeks and sacrificed after 30 weeks from the first urethane injection. Representative images **(O)** and number **(P)** of neoplastic lesions of urethane-treated *Stk24*$^{h/h}$ and WT mice were shown. Each dot represents a biological sample.

Results represent at least two independent experiments and are presented as mean ± SEM. ns, no significant difference. *P<0.05, **P<0.01, ***P<0.001, ****P<0.0001. P values of tumor weight in **M and N** and tumor number in **P** were calculated by unpaired Student's *t*-tests. P values were calculated by two-way ANOVA in **C, E, G, I, K and** tumor growth curves of **M and N**.

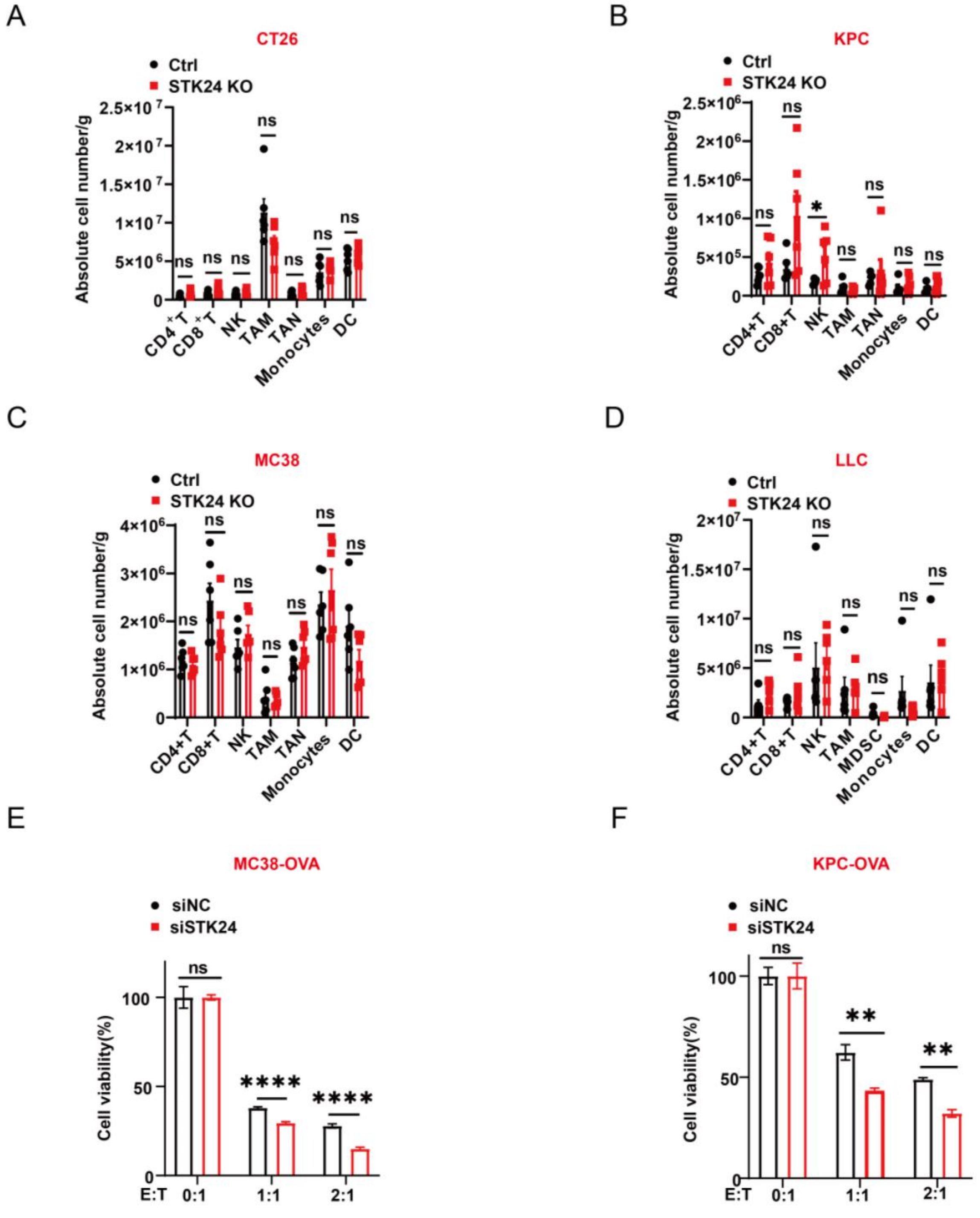

Figure S3. STK24 deficiency promotes the infiltration and activation of CD8$^+$ T and NK cells.

(A-D) Statistical analysis of various tumor-infiltrating immune cells per gram of tumor

tissues from *Ctrl* or *Stk24* KO CT26 **(A)**, KPC **(B)**, MC38 **(C)**, and LLC **(D)** tumor cells subcutaneously inoculated mice models determined by flow cytometry. Each dot represents a biological sample.

(E-F) Quantitative estimates of the number of control or Stk24-knockdown MC38-OVA **(E)** and KPC-OVA**(F)** tumor cells remaining after 48 hours of incubation with activated OT1 cells. E: T is the ratio of T cells and tumor cells. n = 3 biologically independent samples per group.

Results represent at least two independent experiments and are presented as mean ± SEM. ns, no significant difference. *P<0.05, **P<0.01, ****P<0.0001.P values were calculated by unpaired Student's *t*-tests.

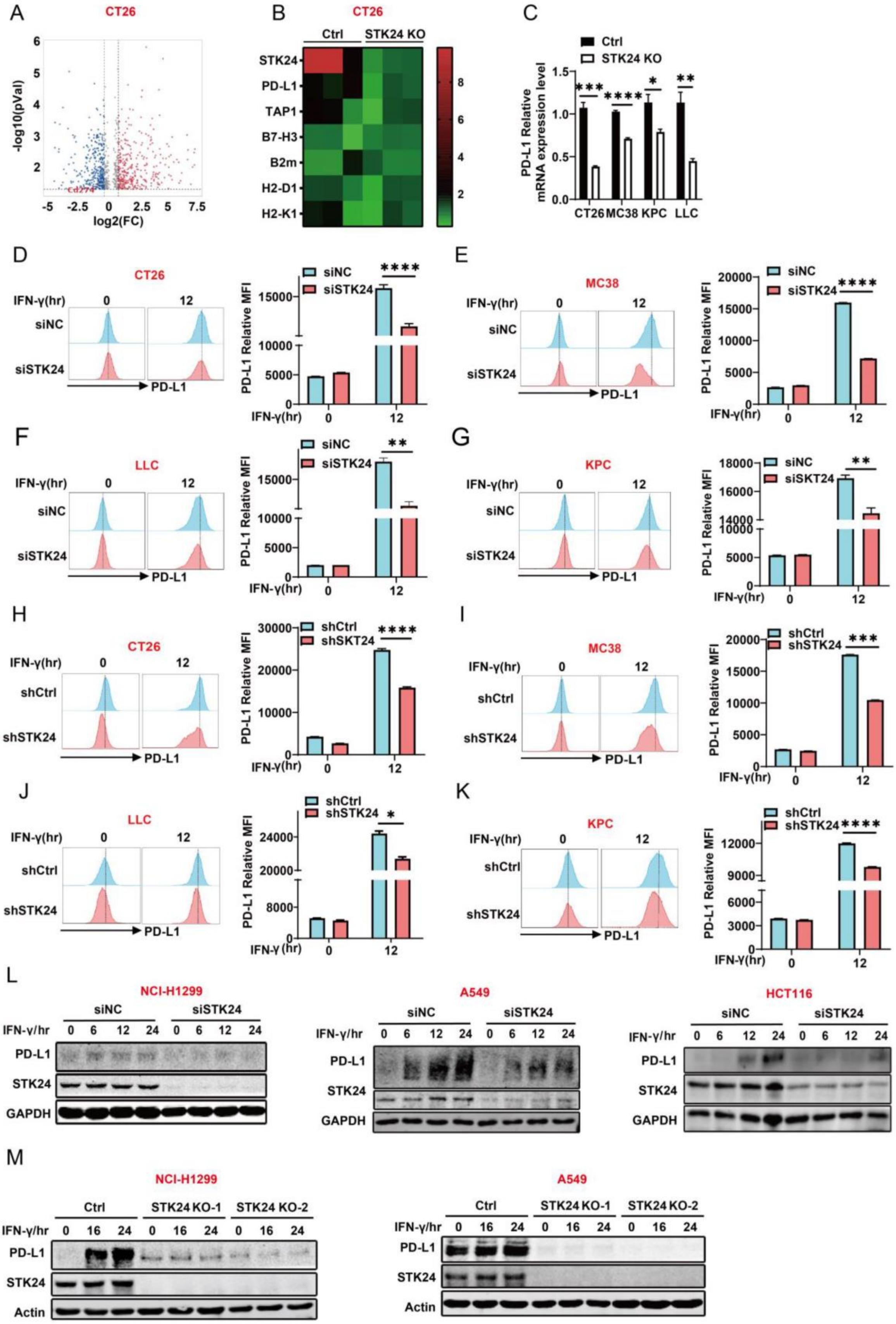
A
CT26
-log10(pVal)
log2(FC)
Cd274
B
CT26
Ctrl
STK24 KO
STK24
PD-L1
TAP1
B7-H3
B2m
H2-D1
H2-K1
C
Ctrl
STK24 KO
PD-L1 Relative mRNA expression level
CT26
MC38
KPC
LLC
D
CT26
IFN-γ(hr)
siNC
siSTK24
PD-L1
PD-L1 Relative MFI
E
MC38
F
LLC
G
KPC
siSKT24
H
CT26
shCtrl
shSTK24
shSKT24
I
MC38
J
LLC
K
KPC
L
NCI-H1299
A549
HCT116
IFN-γ/hr
STK24
GAPDH
M
NCI-H1299
A549
Ctrl
STK24 KO-1
STK24 KO-2
Actin

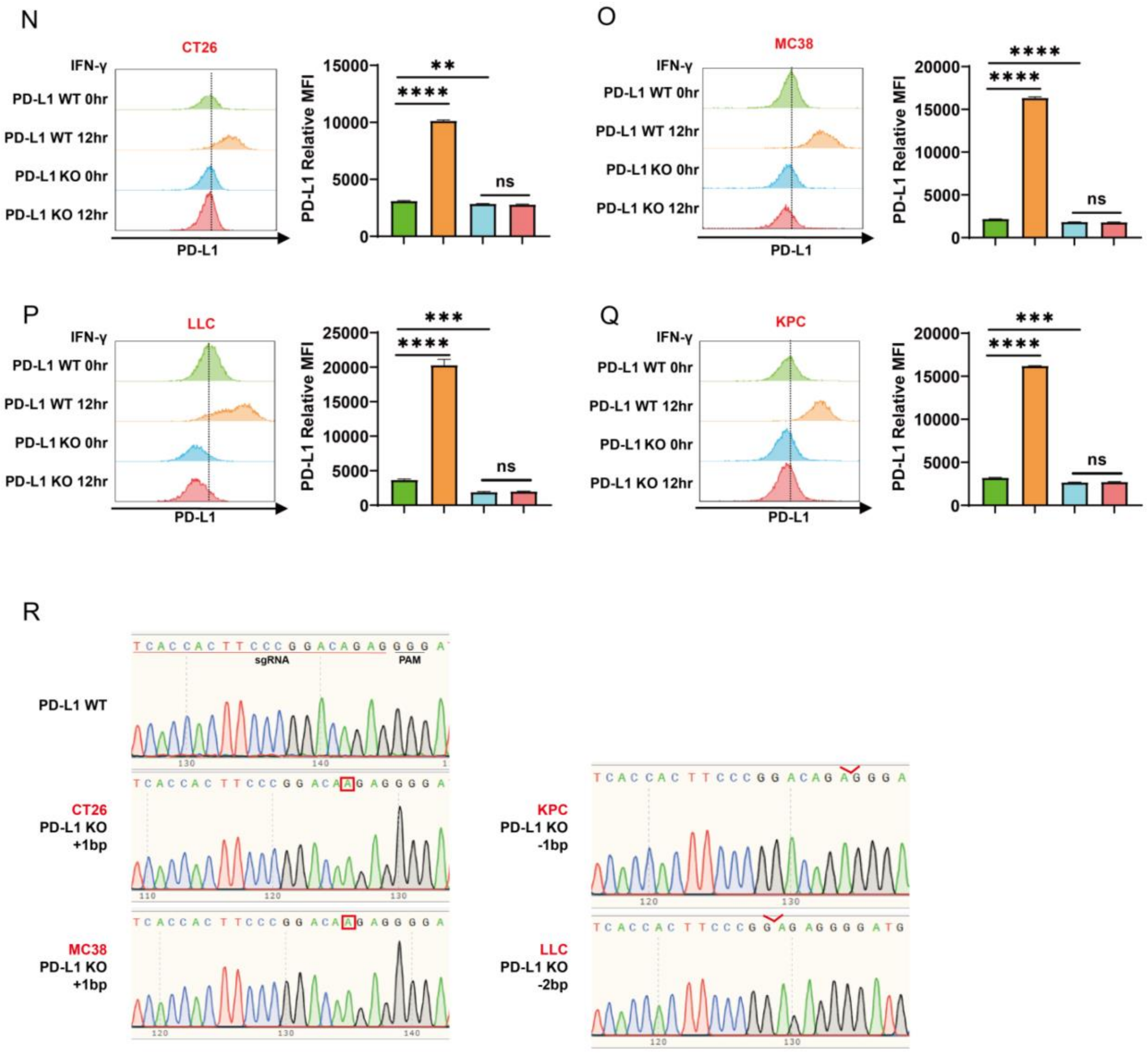

Figure S4. STK24 downregulation in tumor cells inhibits the expression of PD-L1.

(A) Volcano plot from RNA-seq analysis of *Ctrl* and *Stk24* KO CT26 tumor cells.

(B) Heat map from RNA-seq analysis of immune checkpoints related gene expression in *Ctrl* and *Stk24* KO CT26 tumor cells.

(C) The expression of PD-L1 mRNA levels in *Ctrl* and *Stk24* KO CT26, MC38, KPC and LLC tumor cells were detected by real-time qPCR. n = 3 technically independent samples per group.

(D-K) STK24 expression was knockdown in mouse tumor cells by transfection of siRNA **(D-G) or** shRNA lentiviruses **(H-K)**, and then the protein expression of PD-L1 on the tumor cell surface was detected with or without IFN-γ (20 ng/ml) treatment at

the indicated times using flow cytometry. n = 3 biologically independent samples per group.

(L) Immunoblot analysis revealed the attenuated IFN-γ-inducible PD-L1 expression in STK24-knockdown NCI-H1299 **(left)**, A549 **(middle)**, and HCT116 **(right)** tumor cells.

(M) The IFN-γ-inducible PD-L1 expression in STK24-knockout NCI-H1299 **(left)** and A549 **(right)** tumor cells generated by CRISPR/Cas9 was analyzed by Immunoblot analysis.

(N-R) The deficiency of *Pd-l1* cells was confirmed by FACS **(N-Q)** and sanger sequence **(R).** n = 3 biologically independent samples per group.

Results represent at least two independent experiments **(C-M)** and are presented as mean ± SEM. ns, no significant difference. *P<0.05, **P<0.01, ***P<0.001, ****P<0.0001. P values were calculated by unpaired Student's *t*-tests.

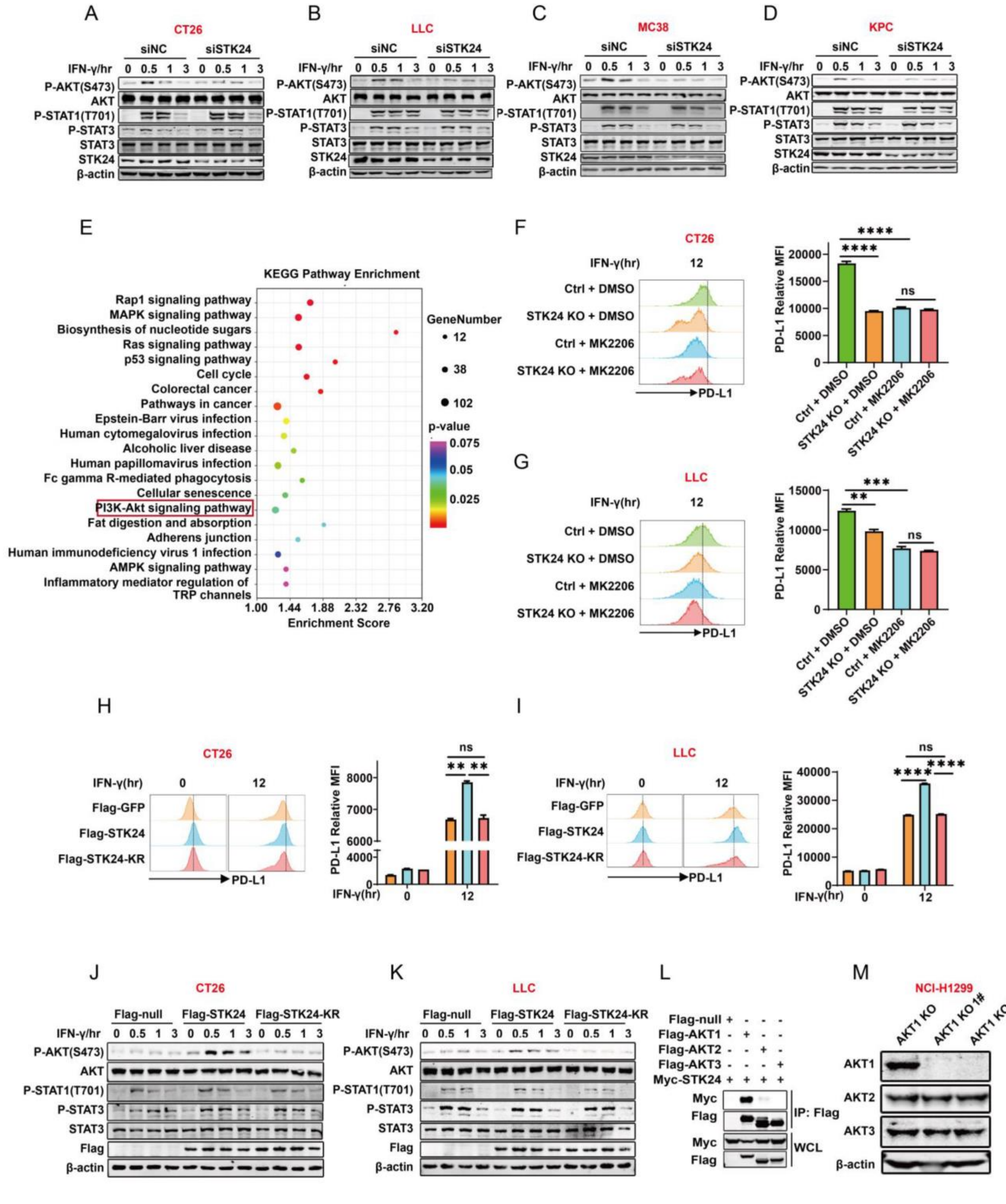
A
CT26
siNC
siSTK24
IFN-γ/hr
P-AKT(S473)
AKT
P-STAT1(T701)
P-STAT3
STAT3
STK24
β-actin
B
LLC
C
MC38
D
KPC
E
KEGG Pathway Enrichment
Rap1 signaling pathway
MAPK signaling pathway
Biosynthesis of nucleotide sugars
Ras signaling pathway
p53 signaling pathway
Cell cycle
Colorectal cancer
Pathways in cancer
Epstein-Barr virus infection
Human cytomegalovirus infection
Alcoholic liver disease
Human papillomavirus infection
Fc gamma R-mediated phagocytosis
Cellular senescence
PI3K-Akt signaling pathway
Fat digestion and absorption
Adherens junction
Human immunodeficiency virus 1 infection
AMPK signaling pathway
Inflammatory mediator regulation of TRP channels
Enrichment Score
GeneNumber
p-value
F
CT26
IFN-γ(hr)
Ctrl + DMSO
STK24 KO + DMSO
Ctrl + MK2206
STK24 KO + MK2206
PD-L1
PD-L1 Relative MFI
G
LLC
H
CT26
Flag-GFP
Flag-STK24
Flag-STK24-KR
I
LLC
J
CT26
Flag-null
Flag-STK24
Flag-STK24-KR
Flag
K
LLC
L
Flag-null
Flag-AKT1
Flag-AKT2
Flag-AKT3
Myc-STK24
Myc
IP: Flag
WCL
M
NCI-H1299
AKT1 KO
AKT1 KO 1#
AKT1 KO 2#
AKT1
AKT2
AKT3

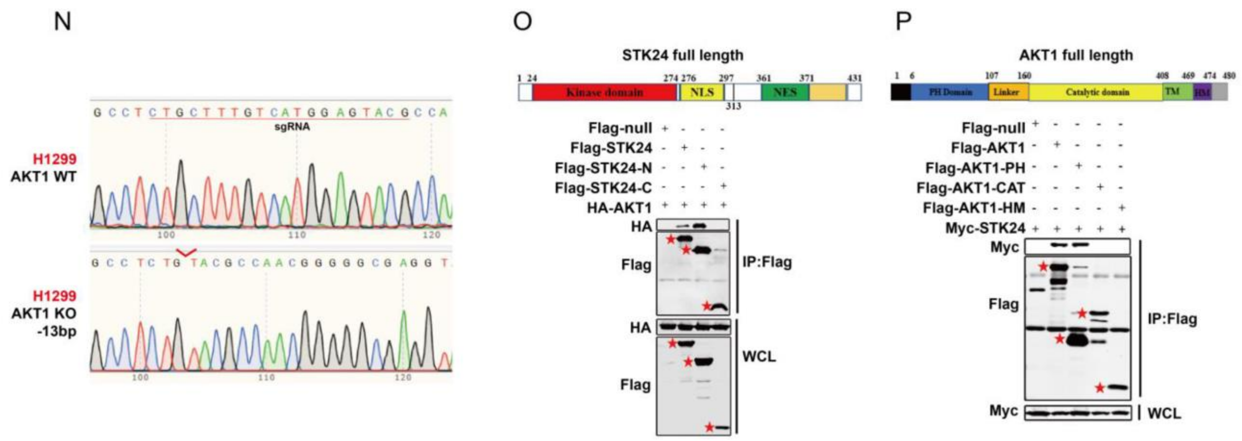

Figure S5. STK24 regulates PD-L1 expression via the activation of the AKT pathway.

(A-D) Immunoblot analysis of AKT signal pathway with the indicated antibodies in STK24-knockout CT26 **(A)**, LLC **(B)**, MC38 **(C)**, and KPC **(D)** cell lines generated by CRISPR/Cas9 treated with IFN-γ (100 ng/ml) for 0.5,1 and 3 hours. n = 3 biologically independent samples per group.

(E) Pathway analysis (KEGG) of RNA-seq in Ctrl and *Stk24* KO LLC tumor cells.

(F-G) FACS and quantification analysis of PD-L1 expression levels of *Ctrl* or *Stk24* KO CT26 **(F)** or LLC **(G)** cell lines pretreated with or without the AKT inhibitor MK2206 (5 μM) for 12 hours and stimulated with IFN-γ for 12 hours. FACS **(left)** and MFI **(right)** of PD-L1^{+} membrane expression was shown. n = 3 biologically independent samples per group.

(H-I) FACS and quantification analysis of PD-L1 expression of IFN-γ treated *Stk24* KO CT26 **(H)** or LLC **(I)** cells rescued with Flag-GFP, Flag-STK24, or Flag-STK24-KR. FACS **(left)** and MFI **(right)** of PD-L1^{+} membrane expression was shown.

(J-K) Immunoblot analysis of AKT signal pathway with the indicated antibodies in *Stk24* KO CT26 **(J)** or LLC **(K)** cells rescued with Flag-null, Flag-STK24, or Flag-STK24-KR treated with IFN-γ (100 ng/ml) for 0.5,1 and 3 hours.

(L) Immunoblot analysis of the interaction between AKT1, AKT2 or AKT3 and STK24 with anti-Flag immunoprecipitated in HEK293T cells. WCL means whole cell lysates.

Flag-null stands for an empty vector.

(M-N) The KO efficiency of *AKT1 KO* NCI-H1299 cells was confirmed by Immunoblot **(M)** and sanger sequence **(N).**

(O) Immunoblot analysis of the interaction between STK24(aa1-431), STK24-N kinase domain (aa1-313) or STK24-C regulator domain (aa314-431) and AKT1 with anti-Flag immunoprecipitated in HEK293T cell line. Flag-null stands for an empty vector.

(P) Immunoblot analysis of the interaction between AKT1 (aa1-480), AKT1 PH domain (aa1-160), AKT1 CAT domain (aa161-408) or AKT1 HM domain (aa409-480), and STK24 with anti-Flag immunoprecipitated in HEK293T cell line.

Results represent at least two independent experiments and are presented as mean ± SEM. ns, no significant difference. **$P<0.01$, ***$P<0.001$, ****$P<0.0001$. P values were calculated by unpaired Student's *t*-tests.

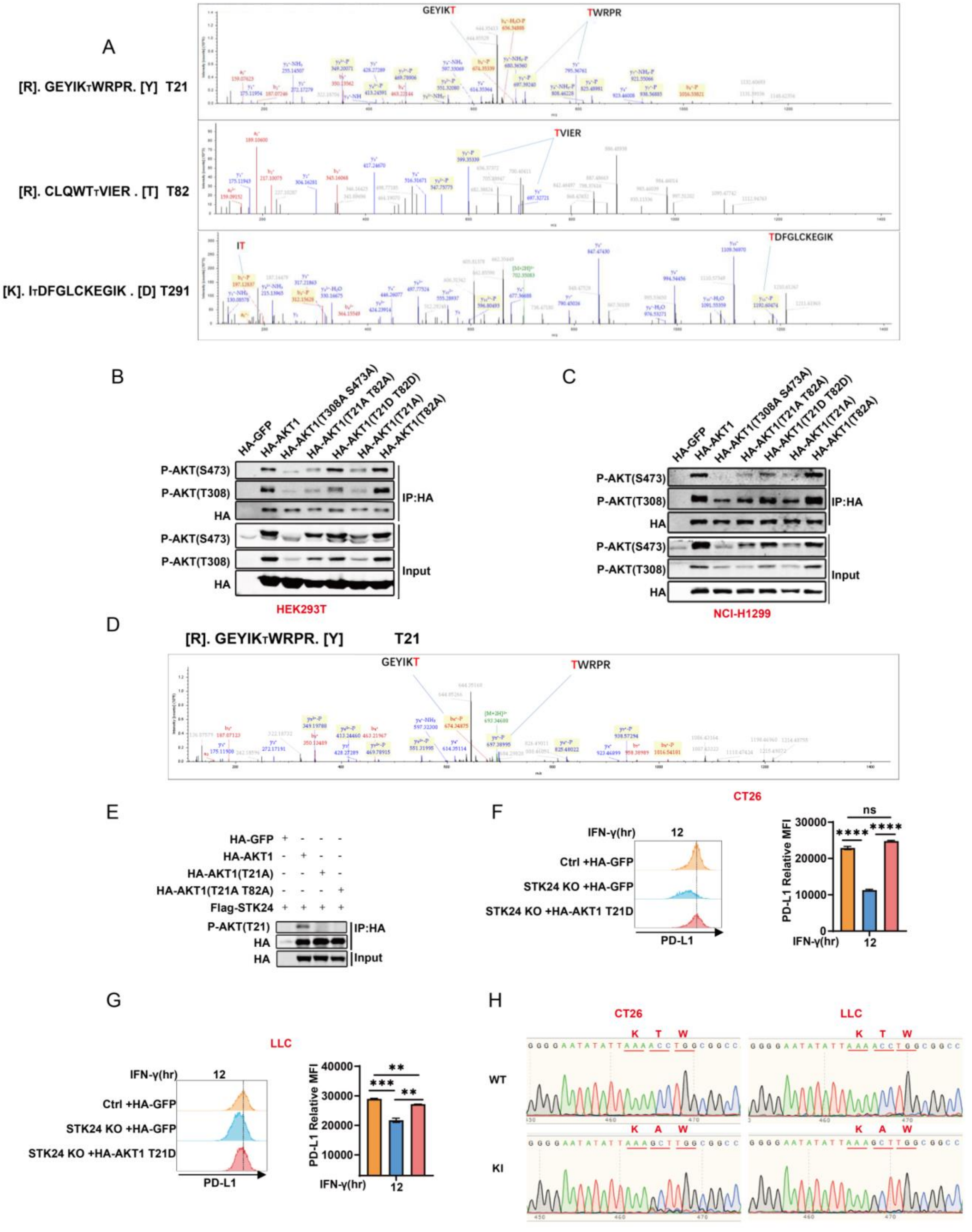
A
[R]. GEYIKTWRPR. [Y] T21
GEYIKT
TWRPR
[R]. CLQWTTVIER . [T] T82
TVIER
[K]. ITDFGLCKEGIK . [D] T291
IT
TDFGLCKEGIK
B
HA-GFP
HA-AKT1
HA-AKT1(T308A S473A)
HA-AKT1(T21A T82A)
HA-AKT1(T21D T82D)
HA-AKT1(T21A)
HA-AKT1(T82A)
P-AKT(S473)
P-AKT(T308)
HA
IP:HA
Input
HEK293T
C
NCI-H1299
D
[R]. GEYIKTWRPR. [Y] T21
E
HA-GFP
HA-AKT1
HA-AKT1(T21A)
HA-AKT1(T21A T82A)
Flag-STK24
P-AKT(T21)
F
CT26
IFN-γ(hr)
12
Ctrl +HA-GFP
STK24 KO +HA-GFP
STK24 KO +HA-AKT1 T21D
PD-L1
PD-L1 Relative MFI
ns
****
G
LLC
***
**
H
K T W
K A W
WT
KI

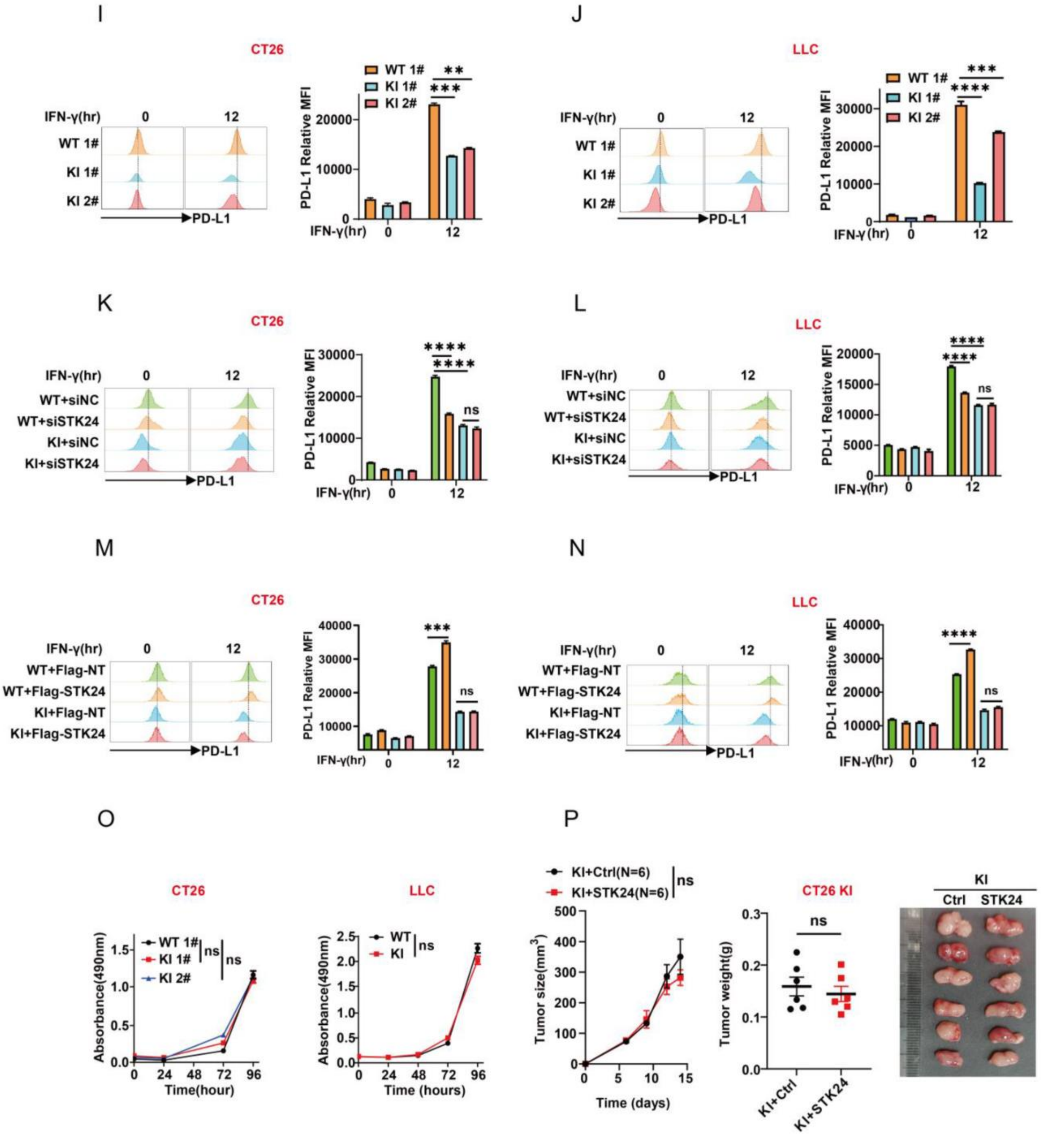

Figure S6. STK24 regulates tumor growth by phosphorylating AKT at Thr21.

(A) The *in vitro* kinase assay reaction mixtures were analyzed by mass spectrometry to identify novel AKT phosphorylation residues directly mediated by STK24. Thr21, Thr82, and Thr291 residues were found phosphorylated by mass spectrometric analysis. **(B-C)** HA-AKT1, or HA-AKT1 mutants, including HA-AKT1-S473A T308A, HA-AKT1-T21AT82A, HA-AKT1-T21DT82D, HA-AKT1-T21A, and HA-AKT1-T82A

was transfected into AKT1-KO HEK293T cells **(B)** or AKT1-KO NCI-H1299 cells **(C)**, and then the phosphorylation of AKT was determined with the indicated antibodies after an anti-HA immunoprecipitation by western blotting.

(D) HA-AKT1 co-expressed with vector or STK24 was immunoprecipitated in HEK293T cells and Thr21 residue was found phosphorylated by mass spectrometric analysis.

(E) Immunoblot analysis of P-AKT (T21) after an anti-HA immunoprecipitation from the lysate of HEK293T cells transfected with Flag-STK24 and HA-AKT1, HA-AKT1-T21A, or HA-AKT1-T21A T82A.

(F-G) AKT1 T21D was overexpressed in *Stk24* KO CT26 **(F)** and LLC **(G)** cells and then the protein expression of PD-L1 on the tumor cell surface was detected with IFN-γ treatment for 12 hours. n = 3 biologically independent samples per group.

(H) Sanger sequence of AKT1 T21A knock-in CT26 and LLC cells.

(I-J) FACS and quantification analysis of PD-L1 expression in WT or KI CT26 **(I)** and LLC **(J)** cell lines treated with or without IFN-γ for 12 hours. n = 3 biologically independent samples per group.

(K-L) STK24 expression was knocked down in mouse WT or KI CT26 **(K)** and LLC **(L)** cells by transfection of siRNA, and then the protein expression of PD-L1 on the tumor cell surface was detected with or without IFN-γ treatment at the indicated time. n = 3 biologically independent samples per group.

(M-N) STK24 was overexpressed in mouse WT or KI CT26 **(M)** and LLC **(N)** cells and then the protein expression of PD-L1 on the tumor cell surface was detected with or without IFN-γ treatment. n = 3 biologically independent samples per group.

(O) The proliferation of the WT and KI CT26 **(left)** or LLC **(right)** cells by serum stimulation.

(P) STK24 stably overexpressed KI CT26 cells (5×10^5) were subcutaneously transplanted into BALB/c mice. Tumor growth curves **(left)**, tumor weight **(middle)**, and representative tumor images **(right)** were shown. Each dot represents a biological sample.

Results represent at least three independent experiments and are presented as mean ± SEM. ns, no significant difference. *P<0.05, **P<0.01, ***P<0.001, ****P<0.0001. P values were calculated by unpaired Student's *t*-tests in **F-G, I-N** and tumor weight of **P** and two-way ANOVA in **O and** tumor growth curves of **P**.

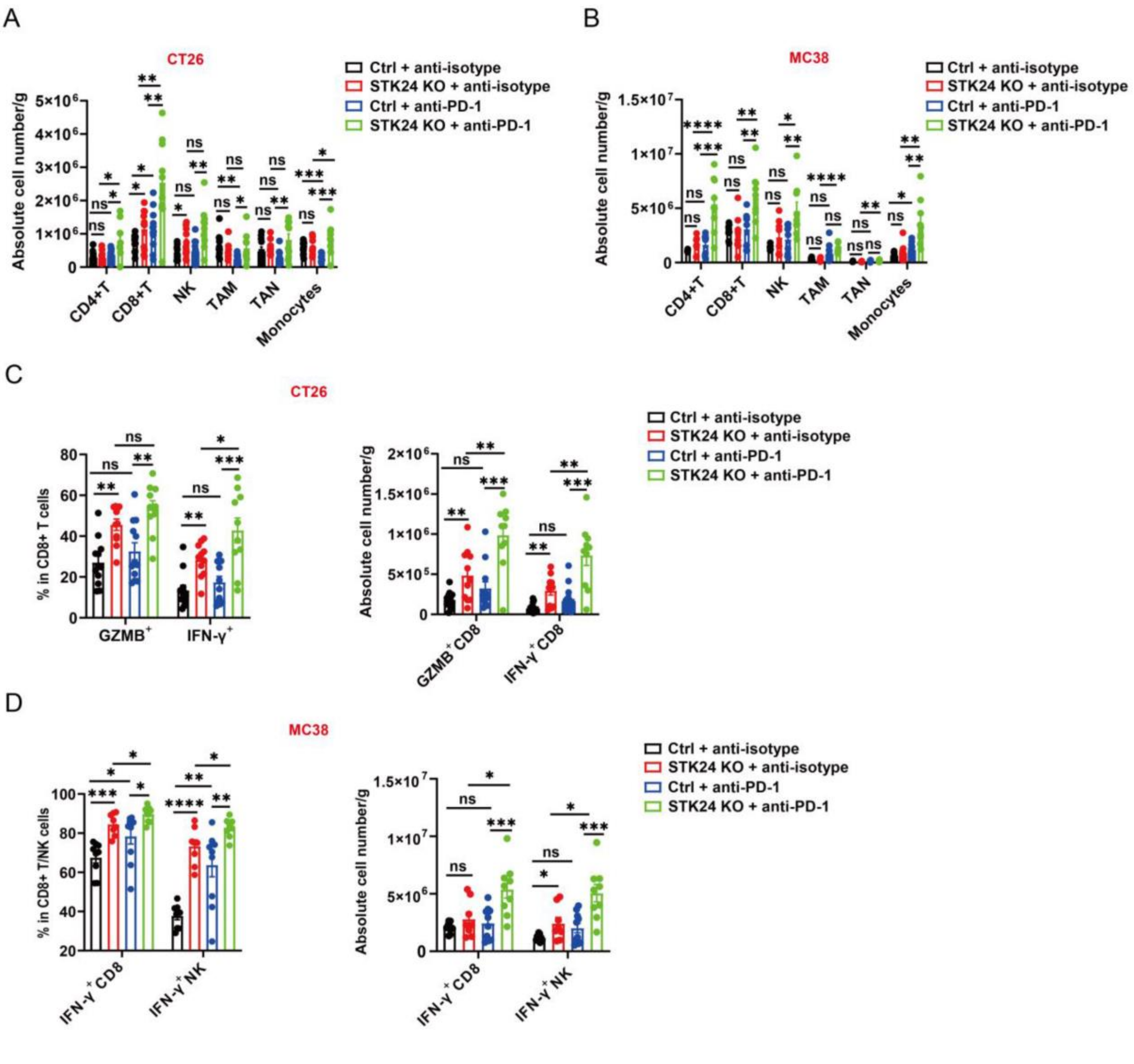

Figure S7. The combination of STK24 deficiency with anti-PD1 therapy boosts the activation of tumor infiltrating lymphocytes (TILs).

(A-B) Statistical analysis of various tumor-infiltrating immune cells per gram of tumor tissues from Ctrl or *Stk24* KO CT26 (A) and MC38 (B) tumor cells subcutaneously inoculated mice models treated with the anti-PD-1 mAb or IgG2α determined by flow

cytometry.

(C-D) Flow cytometry analysis of expression of IFN-γ and Granzyme-B by tumor infiltrating lymphocytes (TILs) from Ctrl and *Stk24* KO CT26 (C) or MC38 (B) tumor cells subcutaneously inoculated mice models treated with the anti-PD-1 mAb or IgG2α Results represent at least two independent experiments and are presented as mean ± SEM. Each dot represents a biological sample. ns, no significant difference. *P<0.05, **P<0.01, ***P<0.001, ****P<0.0001. P values were calculated by unpaired Student's *t*-tests.

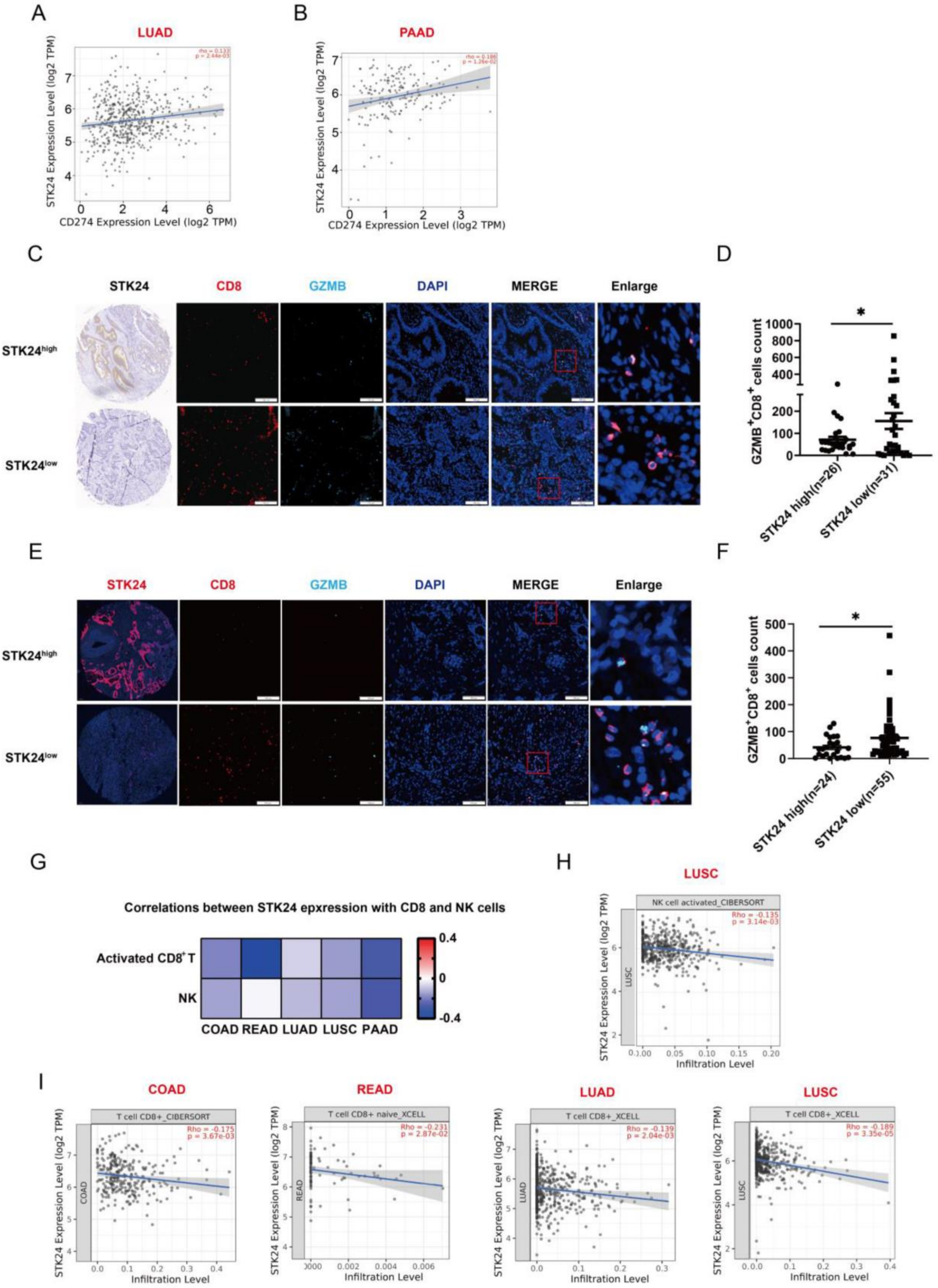
A
LUAD
STK24 Expression Level (log2 TPM)
CD274 Expression Level (log2 TPM)
B
PAAD
STK24 Expression Level (log2 TPM)
CD274 Expression Level (log2 TPM)
C
STK24
CD8
GZMB
DAPI
MERGE
Enlarge
STK24high
STK24low
D
GZMB+CD8+ cells count
STK24 high(n=26)
STK24 low(n=31)
*
E
STK24
CD8
GZMB
DAPI
MERGE
Enlarge
STK24high
STK24low
F
GZMB+CD8+ cells count
STK24 high(n=24)
STK24 low(n=55)
*
G
Correlations between STK24 epxression with CD8 and NK cells
Activated CD8+ T
NK
COAD READ LUAD LUSC PAAD
H
LUSC
NK cell activated_CIBERSORT
Rho = -0.135
p = 3.14e-03
STK24 Expression Level (log2 TPM)
Infiltration Level
I
COAD
T cell CD8+_CIBERSORT
Rho = -0.175
p = 3.67e-03
READ
T cell CD8+ naive_XCELL
Rho = -0.231
p = 2.87e-02
LUAD
T cell CD8+_XCELL
Rho = -0.139
p = 2.04e-03
LUSC
T cell CD8+_XCELL
Rho = -0.189
p = 3.35e-05
STK24 Expression Level (log2 TPM)
Infiltration Level

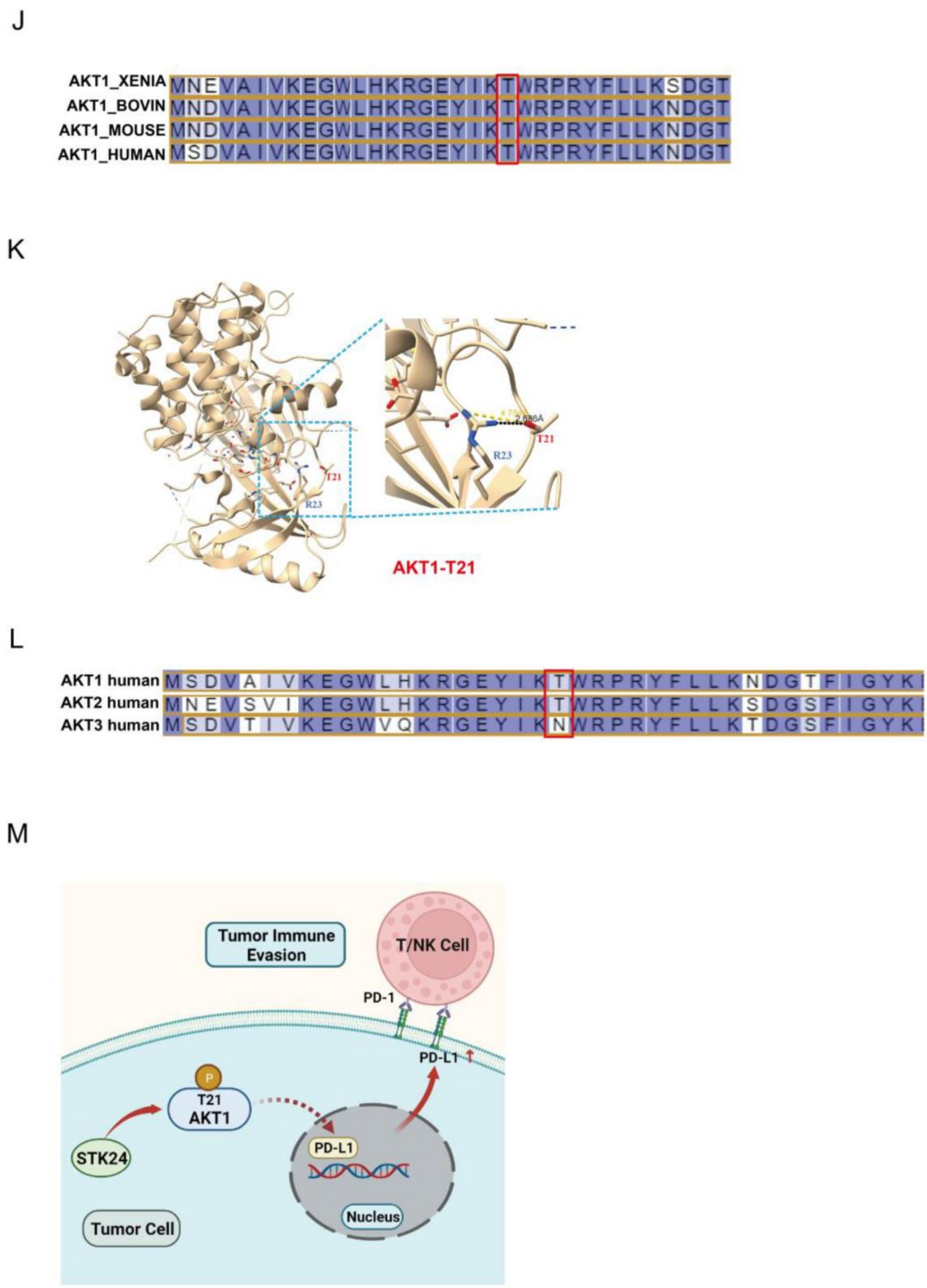

Figure S8. STK24 expression is negatively correlated with immune cell infiltration in colorectal, lung, and pancreatic cancer patients.

(A-B) Correlation analysis of STK24 expression with CD274 expression in tumor tissues of LUAD **(A)** and PAAD **(B)** in the TIMER database.

(C) TMA based-Immunofluorescence staining analysis of $GZMB^{+}CD8^{+}$ T cells in tumors of CRC patients. Scale bars, 100 μm.

(D) Quantitative analysis of the infiltration of $GZMB^{+}CD8^{+}$ T cells in the STK24 high and STK24 low CRC groups.

(E) TMA based-immunofluorescence staining analysis of $GZMB^{+}CD8^{+}$ T cells in tumors of PAAD patients. Scale bars, 100 μm.

(F) Quantitative analysis of the infiltration of $GZMB^{+}CD8^{+}$ T cells in the STK24 high and STK24 low PAAD patients' groups.

(G) Correlation analysis of STK24 expression with the infiltration of $CD8^{+}$ T cells and NK cells in tumor tissues of COAD, READ, LUAD, LUSC, and PAAD in the TISIDB database. The red color indicates a positive correlation and the blue color indicates a negative correlation

(H-I) Correlation analysis of STK24 expression with NK cell infiltration in tumor tissues of LUSC **(H)** and $CD8^{+}$ T cells infiltration in tumor tissues of COAD, READ, LUAD, and LUSC **(I)** in the TIMER database.

(J) Alignment of AKT1 from different species with the position of Thr21 highlighted in the red box.

(K) Structural comparison of the AKT1 (PDB: 7NH5) with T21.The distances of T21 and R23 was less than 5Å and its interactions with R23 are highlighted.

(L) Alignment of human AKT1, AKT2 and AKT3 with the position of Thr21 highlighted in the red box.

(M) STK24 orchestrates tumor immune evasion via phosphorylating AKT1 and promoting PD-L1 expression. Created with BioRender.com.

Results are presented as mean ± SEM. *$P<0.05$.P values were calculated by unpaired Student's *t*-tests in **D and F**.

Reference

[1] Q. Qin, J. N. Shou, M. J. Li, M. D. Gu, Z. X. Meng, P. L. Xu, H. Meng, X. J. Wang, *Cell Rep* **2021**, *35* (8), https://doi.org/ARTN 109161
10.1016/j.celrep.2021.109161.

[2] F. A. Ran, P. D. Hsu, J. Wright, V. Agarwala, D. A. Scott, F. Zhang, *Nat Protoc* **2013**, *8* (11), 2281, https://doi.org/10.1038/nprot.2013.143.
